# A clubroot pathogen effector targets cruciferous cysteine proteases to suppress plant immunity

**DOI:** 10.1080/21505594.2021.1968684

**Published:** 2021-09-13

**Authors:** Edel Pérez-López, Md Musharaf Hossain, Yangdou Wei, Christopher D. Todd, Peta C Bonham-Smith

**Affiliations:** a Department of Biology, University of Saskatchewan, Saskatoon, Canada; b Department of Plant Sciences, University Laval, Criv, Quebec City, Canada

**Keywords:** Cysteine protease inhibitor, papain-like cysteine proteases (plcps), apoplast, clubroot, plant defense, *plasmodiophora brassicae*

## Abstract

Plant pathogen effector proteins are key to pathogen virulence. In susceptible host Brassicas, the clubroot pathogen, *Plasmodiophora brassicae*, induces the production of nutrient-sink root galls, at the site of infection. Among a list of 32 *P. brassiae* effector candidates previously reported by our group, we identified SSPbP53 as a putative apoplastic cystatin-like protein highly expressed during the secondary infection. Here we found that SSPbP53 encoding gene is conserved among several *P. brassicae* pathotypes and that SSPbP53 is an apoplastic protein able to directly interact with and inhibit cruciferous papain-like cysteine proteases (PLCPs), specifically Arabidopsis XYLEM CYSTEINE PEPTIDASE 1 (*At*XCP1). The severity of clubroot disease is greatly reduced in the Arabidopsis *xcp1* null mutant (*At*Δ*xcp1*) after infection with *P. brassicae* resting spores, indicating that the interaction of *P. brassicae* SSPbP53 with XCP1 is important to clubroot susceptibility. SSPbP53 is the first cystatin-like effector identified and characterized for a plant pathogenic protist.

## Introduction

Clubroot is caused by the obligate biotrophic, soil-borne, parasitic protist *Plasmodiophora brassicae* member of the order Rhizaria [1] that can infect most plants in the *Brasicaceae* family, including Arabidopsis. A *P. brassicae* primary infection of a susceptible host plant, through primary zoospores intrusion of root hairs, leads to the production of secondary zoospores that, upon secondary infection of cortical cells, results in the reprogramming of phloem and xylem development and the establishment of nutrient-sink galls to support the production of new resting spores [2,3]. To complete the life cycle *P. brassicae* needs to escape or down-regulate the host plant microbe-associated molecular patterns (MAMPs)-triggered immunity (MTI) [4]. Many biotrophic and necrotrophic plant pathogens regulate MTI by manipulating plant salicylic acid (SA) metabolism [5–7]. Similarly, *P. brassicae* produces a methyltransferase, *Pb*BSMT, that methylates SA, benzoic and anthranilic acids, thereby reducing SA content in the host cell and attenuating plant defense responses [8,9]. A key component of the sophisticated plant immune system are papain-like cysteine proteases (PLCPs). Aimed at the detection of MAMPS and the induction of the plant defense response, PLCPs bind to and degrade pathogen effector proteins [10,11]. Recent reports suggest that PLCPs are also involved in the induction of systemic immunity and the promotion of cell death during an infection [12,13].

To counter PLCP activity, many evolutionary unrelated biotrophic pathogens produce PLCP inhibitors during colonization of the plant host [14–21]. For example, Avr2 from the extracellular pathogen *C. fulvum*, is able to inhibit the Arabidopsis PLCPs CPR1, XCP1, and XCP2 as well as tomato Rcr3 and Pip1 [22]; EPI1 and EPIC2B from *Phytophthora infestans* are inhibitors of the tomato PLCP PIP1 [23]; SDE1 from the Huanglongbing (HLB) pathogen *Candidatus* Liberibacter asiaticus inhibits citrus RD19a, RD21a, and SAG12 PLCPs [21]; and Pit2 from *Ustilago maydis* inhibits CP2, a maize PLCP member of the aleurain like cysteine protease family [19], suggesting a key role for PLCPs in the plant immune response and a key target by pathogens.

Using genome-wide transcriptomic analysis we have previously identified, small secreted *P. brassicae* protein 53 (SSPbP53: PBRA_008207), to be a cystatin-like PLCP inhibitor, with a functional signal peptide, that is over-expressed during secondary infection and subsequent gall formation in *P. brassicae* infected Arabidopsis roots [24]. In this study, further analyses of SSPbP53 during clubroot disease development show that it inhibits PLCP activity from six susceptible cruciferous plant hosts, with a clear affinity for the xylogenesis-related XCP1 in Arabidopsis. This characterization of the *P. brassicae* effector protein and its corresponding plant PLCP provides a possible target to be exploited for clubroot management and the generation of increased resistant *Brassicaceae* varieties. This is the first description of such a mechanism for a plant pathogenic member of the order Rhizaria.

## Materials and methods

### Plant material and growth conditions

Arabidopsis, rapeseed (*B. napus*), arugula (*Eruca sativa*), broccoli (*B. oleracea* var. *italica*), cabbage (*B. oleracea* var. *oleracea*), wild mustard (*B. kaber*), and *Nicotiana benthamiana* seeds, disinfected with 70% ethanol, 95% ethanol, 10% bleach and sterile water, were sown on agar (1%, w/v) plates containing 1/2 Murashige and Skoog (MS) (Sigma-Aldrich, CAD) salts with 1% sucrose. Plates were sealed with parafilm, placed at 4°C for 4 days and then transferred to a growth chamber (Conviron E8, CMP6050 control system; 100 μmol photons m^2^ s^−1^; 16 h/8 h light/dark cycle; 22°C) for germination and growth. The Arabidopsis mutants *At*Δ*rd19* (SALK_031088) and *At*Δ*xcp1* (SALK_084789) were obtained from the Arabidopsis Biological Resource Center (TAIR, Ohio State University), while the *At*Δ*rd21* mutant was kindly provided by Prof. Robert Fluhr [25]. All homozygous mutants were confirmed by RT-PCR to ensure the lack of expression of their respective gene.

### Infection and disease indexing

Ten-day old cruciferous seedlings were transferred to Sunshine Mix #4 soil (Sun Gro Horticulture Inc., BC) with four plants in each “square pot” (3.5 x 3.5 inch) of an 18-pot sheet. Seedlings were allowed to acclimatize in these pots for four additional days before 400 uL of 5 × 10^7^ resting spores mL^−1^ of a Saskatchewan field isolate of *P. brassicae* pathotype 3 H, was applied to each plant where the stem entered the soil. Control plants were inoculated with 400 μL of distilled water and grown in separate trays in the same growth chamber.

Disease index (DI) was determined for *At*Col-0, *At*Δ*xcp1, At*Δ*rd19* and *At*Δ*rd21* infected plants at 21 dpi as previously described [26]. Root infection was assessed on a 0–4 scale where 0, no symptoms; 1, small galls mainly on lateral roots; 2, small galls on the main and lateral roots; 3, medium-sized galls with possible negative effect on plant growth and 4, severe galls on both main and lateral roots, deformed roots and impaired growth [26]. The experiment, repeated three times, was arranged in a completely randomized design with the DI of infected plants determined from 68 plants per experimental unit.

### Spore quantification

Spore production was determined for clubroot infected *At*Col-0 and *At*Δ*xcp1* plants at 21 dpi. The soil in each of the 51 square pots for each plant line was air-dried at room temperature (21 ± 2°C), thoroughly mixed, weighed and 0.5 g of soil was collected for resting spore extraction [27]. Soil was mixed, using a blender, with 20 mL of sterile distilled water for 1 min and filtered through eight layers of cheesecloth. The pass-through was centrifuged at 3900 *g* (Allegra^TM^ 25 R Centrifuge) for 15 min and the supernatant was discarded. The pellet was suspended in 6 mL of 50% (w/v) sucrose by vortex for 2 min and then centrifuged for 5 min at 1700 *g*. The supernatant was transferred to a 50 mL tube, brought to 50 mL with sterile distilled water, mixed with a vortex and centrifuged at 3900 *g* for 15 min. Pellets were resuspended in 5 mL of sterile distilled water, vortexed and centrifuged and the supernatant was again discarded and the pellet suspended in 2 mL of sterile distilled water. Spores were counted using a hemocytometer and expressed as the number of spores per plant.

### Structural analysis of SSPbP53

Model structures for SSPbP53 and CHIKCYS were created using SWISS-MODEL [28,29], based on crystal structures in the PDB database with the highest structural similarity to each query protein. The models of SSPbP53 and CHIKCYS were modeled using PyMOL [30].

### Pathotype SSPbP53 analysis

To amplify *SSPbP53*, genomic DNAs (gDNA) of *Plasmodiophora brassicae* pathotype 3 H (Pb3H) obtained from Dr. Gary Peng (AAFC-Saskatoon Research Center) and single spore isolates SACAN-SS_3_ (Pb2), ORCA-SS_3_ (Pb5), AbotJE-SS_3_ (Pb6), and CDCN-SS_1_ (Pb8) [31], were used. PCR amplification used Phusion High-Fidelity DNA Polymerase (Thermo Fisher Scientific, CAD) in a 50 μL final volume containing 300 nM of each primer (gSSPbP53F/gSSPbP53R, Supplementary Table S1) and 2 to 4 ng of gDNA. The 530 bp amplicon generated for each pathotype was purified using GeneJET PCR Purification Kit (Fermentas Life Science, CAD) and directly sequenced with the amplification primers (Eurofins Scientific, CAD).

### Plasmid construction

For plasmid construction, the Gateway cloning system (Thermo Fisher Scientific, CAD) was used following the manufacturer´s recommendations. All plasmids used and generated in this study are listed in Supplementary Table S2.

To amplify all of the coding sequences cloned in this study, total RNA was extracted from Pb3 infected (when pathogen cDNA needed), or uninfected Arabidopsis Col-0 roots using a Trizol based extraction method [32]. cDNA was synthesized from 2 ug of total RNA using the QuantiTect® Reverse Transcription Kit (Qiagen, Canada) following the manufacturer’s recommendations. Each cDNA was used as a template to amplify *SSPbP53* (*SSPbP53* is minus the signal peptide sequence and with the stop codon at the C-terminus, PBRA_008207), *AtSAG12*-cys (AT5G45890), *AtRD19*-cys (AT4G39090), *At*RD21-cys (AT1G47128), *AtAALP*-cys (AT5G60360), *AtCATHB3*-cys (AT4G01610), *AtXBCP3*-cys (AT1G09850), and *AtXCP1*-cys (AT4G35350) coding sequences, adding the *att*B1 and *att*B2 recombination sites at the 5ʹ and 3ʹ ends, respectively, with the designated primer pairs (Supplementary Table S1). The *att*B-flanked DNA fragments were cloned into pDON R™/Zeo (Thermo Fisher Scientific, CAD) using BP Clonase™ (Thermo Fisher Scientific, CAD) following the manufacturer´s recommendations, generating entry clones.

To study the interaction between SSPbP53 and *At*PLCPs through yeast two-hybrid (Y2H) assay, the coding sequences previously cloned into pDON R™/Zeo were cloned into pDEST32 (Thermo Fisher Scientific, CAD) and pDEST22 (Thermo Fisher Scientific, CAD), respectively, using LR Clonase™ (Thermo Fisher Scientific, CAD) following the manufacturer´s recommendations. To express SSPbP53 *in planta*, the coding sequence was cloned under the control of the CaMV 35S promoter into pEarlyGate100 (without fluorescent tag) and pEarlyGate103 (with N-terminus GFP) (Thermo Fisher Scientific, CAD), using LR Clonase™ (Thermo Fisher Scientific, CAD) and the pDONZeo_SSPbP53 entry vector following the manufacturer´s recommendations. Constructs generated are listed in Supplementary Table S2. To use as an empty vector (EV) control, SSPbP53 signal peptide sequence followed by a C-terminal stop codon was cloned in frame into pEarlyGate103 to remove the toxic *ccdB* selection marker.

To obtain His-SSPbP53, the coding sequence was cloned into pDEST17 using LR Clonase™ (Thermo Fisher Scientific, CAD) and the SSPbP53_pDONR/Zeo entry vector following the manufacturer´s recommendations. The same procedure was followed to obtain GST-tagged *At*AALP-cys, *At*CATHB3-cys, *At*XBCP3-cys, and *At*XCP1-cys, from coding sequences that were previously cloned into their corresponding entry vector and transferred into pDEST15 using LR Clonase™ (Thermo Fisher Scientific, CAD) following the manufacturer´s recommendations. To use as an empty vector (EV) control, SSPbP53 signal peptide sequence followed by a C-terminal stop codon was cloned in frame into pDEST15 to remove the toxic *ccdB* selection marker.

To obtain the His-SSPbP53^ΔL1^ mutant (the deletion of loop 1), the coding sequence for SSPbP53^ΔL1^ was synthesized (GenScript, USA) and cloned into pET-14b (GenScript, USA). The same procedure was followed to obtain His-*At*XCP1-cys.

### Yeast two-hybrid (Y2H) assay

The recombinant plasmid pDEST32_SSPbP53 was transformed into the *Saccharomyces cerevisiae* strain AH109 to generate the bait. The pDEST22_ *At*SAG12-cys, _*At*RD19-cys, _*At*RD21-cys, *At*AALP-cys, _*At*CATHB3-cys, _*At*XBCP3-cys, and *AtXCP1*-cys plasmids were transformed into the bait and growth into on SD-2 media to confirm yeast transformation. The interaction was assessed through selection on SD-3 media. Yeast transformed with the empty vectors served as negative controls. The experiments were repeated three times with similar results.

### Production of recombinant proteins

For protein expression, recombined pDEST15 and pDEST17 vectors were used to transform *E. coli* BL2E cells (Thermo Fisher Scientific, CAD). *E. coli* transformants carrying the respective expression plasmids (Supplementary Table S2) were grown in 50 mL LB medium supplemented with ampicillin (100 μg mL^−1^) at 37°C, 200 rpm for 3 h. Protein expression was induced by the addition of 20% arabinose to a final concentration of 0.02% and growth was continued at 37°C, 200 rpm for 2 h. After centrifugation at 3000 *g* for 15 min, cell pellets were lysed using a sonicator VirTis equipped with a microtip (VirSonic, USA). Pellets expressing GST tagged *AtAALP*-cys, *AtCATHB3*-cys, *AtXBCP3*-cys, and *AtXCP1*-cys were resuspended in 8 mL B-PER^TM^ bacterial cell lysis reagent (Thermo Fisher Scientific, CAD), sonicated on ice using six 10-second bursts at high intensity with a 10-second cooling period between each burst and the lysate was centrifuged at 3000 *g* for 15 min. The supernatant was used in *in vitro* pull-down assays (see below). Pellets containing His-SSPbP53, His-SSPbP53^ΔL1^, and His-*At*XCP1-cys were resuspended in 8 mL of guanidinium lysis buffer, sonicated as described above, and purified with Ni-NTA agarose (Thermo Fisher Scientific, CAD) through hybrid conditions following the manufacturer´s recommendations. Protein concentration was measured using a Qubit protein assay kit (Thermo Fisher Scientific, CAD).

#### In vitro *inhibition assay*

The papain inhibition assay was performed as previously described [21]. Briefly, fluorescein-labeled casein (FITC-casein, Pierce™ fluorescent protease assay kit) (Thermo Fisher Scientific, CAD) was used as substrate. Recombinant His-SSPbP53, protease inhibitor E-64 (Thermo Fisher Scientific, CAD), or BSA (Sigma-Aldrich, CAD) (100 and/or 500 nM), were mixed with 100 μg mL^−1^ papain (Sigma-Aldrich, CAD) in 96-well plates (Thermo Fisher Scientific, CAD) in the dark. Papain was also mixed with MES buffer as the negative control. After 1 h at room temperature in the dark, fluorescence was measured using a microplate fluorometer Fluoroskan Ascent® (Thermo Fisher Scientific, CAD) at 485/538 nm excitation/emission, with a gain value of 40, following the manufacturer´s recommendations. Each experiment was repeated three times with similar results.

For SSPbP53 and SSPbP53^ΔL1^ inhibition of *At*XCP1, a similar experiment using fluorescein-labeled casein as substrate and the recombinant proteins at 500 nM was performed. Fluorescence was measured using a microplate fluorometer EPOCH 2 (BioTek, USA) as described above. This experiment was repeated three times.

### Western blot analysis

Proteins were separated by 15% SDS-PAGE and transferred to 0.2 μm PVDF membranes (Bio-Rad, CAD) for 1 h at 0.4 V cm^−1^ in a Mini-PROTEAN® Tetra Cell (Bio-Rad, CAD). His-tagged proteins were detected using 1:1000 diluted monoclonal anti-polyHis-HRP conjugate (Sigma-Aldrich, CAD). GFP and GST tagged proteins were visualized with 1:1500 diluted monoclonal anti-GFP and anti-GST-HRP conjugates, respectively (Santa Cruz Biotechnology, USA). Biotinylated proteins were detected with 1:2000 diluted HRP-conjugated Streptavidin (Thermo Fisher Scientific, CAD). Membranes were blocked with 5% skim-milk (BD Difco, CAD) and HPR activity was detected using SuperSignal™ West Pico PLUS Chemiluminescent Substrate (Thermo Fisher Scientific, CAD) with a ChemiDoc™ Imaging System (Bio-Rad, CAD) able to detect the chemiluminiscent signal.

### Activity-based protein profiling

Apoplastic fluid was obtained following the previously described protocol of de Wit and Spikman [33]. Papain at 100 μg mL^−1^ (Sigma-Aldrich, CAD), Arabidopsis, rapeseed, arugula, broccoli, cabbage, wild mustard, and *N. benthamiana* apoplastic fluids were pretreated with either MES buffer (negative control), 100 nM E-64 (positive control), or 500 nM SSPbP53. For rapeseed cotyledons expressing SSPbP53, apoplastic fluids were pretreated only with MES buffer or E-64 (100 nM). Following 1 h pretreatment at room temperature, the samples were incubated with a final concentration of 2.5 μM DCG-04 (Syntides, CHN) for 3 h at room temperature, followed by acetone precipitation of the proteins. Precipitated proteins were re-suspended in 50 μL of 50 mM Tris buffer (pH 6.4) and enriched using streptavidin magnetic beads (Thermo Fisher Scientific, CAD). Resuspended proteins were incubated with 25 μL streptavidin magnetic beads at room temperature for 1 h, washed twice with 1% SDS, and eluted by heating for 5 min at 95°C in Laemmli sample buffer with 13% β-mercaptoethanol as previously described [21]. Biotinylated proteins were detected by western blot as described above. Each experiment was repeated three times with similar results. Two-week old rapeseed cotyledons were infiltrated with *A. tumefaciens* containing untagged SSPbP53 to assess SSPbP53 inhibitory ability.

### Microscopy

Two-week-old rapeseed cotyledons and 4-week-old *N. benthamiana* leaves were infiltrated with 0.3 OD_600_ *A. tumefaciens* GV3101 (pMP90) containing pEG103_SSPbP53-GFP. Fluorescence was observed using a Zeiss LSM-510 confocal microscope with a PL APO 40×/1.3 water-dipping objective and 488/500-530 nm excitation/emission after no more than 2 days post infiltration. To assess SSPbP22 localization, 4-week-old *N. benthamiana* leaves were infiltrated with 0.3 OD_600_ *A. tumefaciens* containing pEG103_SSPbP22-GFP [24] and fluorescence was observed using a Zeiss Axioskop 2 plus fluorescence microscope and 488/500-530 nm excitation/emission after 3 days post infiltration.

Roots of *A. thaliana* Col-0 and *xcp1* Pb+ at 20 dpi and *xcp1* Pb- were fixed using FAA solution (formaldehyde 37%: ethanol 99%: acetic acid, 10%:50%:5% + 35% water) and immobilized in parafilm wax for 5 μm sectioning using a Microm microtome.

### Gene expression analysis by qRT-PCR

cDNAs for *P. brassicae* infected and non-infected root tissue at 14, 21 and 28 dpi were diluted 1:80 (v:v) in RNase-free water and used to detect the expression of Arabidopsis PLCP genes. Real-time qPCR was performed using SsoAdvanced™ Universal SYBR® Green Supermix (Bio-Rad, Canada) in a 20 μL final volume containing 300 nM of each primer, and 2 μL of cDNA diluted 1:5 (v:v) in RNase-free water. Amplification was carried out using a C1000 thermocycler base with a CFX96 real-time system (Bio-Rad) and reactions were quantified using BioRad CFX manager software (v.3.1). Each amplification used three technical replicates, the results of which were averaged to give the value for a single biological replicate. Three biological replicates were prepared for each time point using material harvested from 12 plants in each replicate, grown on three separate occasions. Results are expressed as LOG2 expression relative to *P. brassicae ELONGATION FACTOR-LIKE* (*PbEFL*, PBRA_001540) expression using the comparative quantification method as previously described [34]. Primers used are presented in Table S2.

#### In vitro *pull-down assays*

Three hundred microliters of *E. coli* cell lysate supernatant of GST tagged *AtAALP*-cys, *AtCATHB3*-cys, *AtXBCP3*-cys, and *AtXCP1*-cys were incubated with 25 μL glutathione resin (Thermo Fisher Scientific, CAD) for 1 h at 4°C and washed with TKET buffer (20 mM Tris-HCl, 200 mM KCl, 0.1 mM EDTA, 0.05% Triton X-100, pH 6.0) as previously described [19]. His-SSPbP53-expressing cell lysate was added to the *At*PLCP-bound resins and incubated for 3 h at 4°C, followed by washing with TKET buffer. Resins were then boiled in 25 μL Laemmli sample buffer and the supernatants were analyzed through western blot using anti-His-HRP and anti-GST-HRP as described above.

### Phylogenetic analysis of host PLCPs

The 32 PLCPs from Arabidopsis (TAIR; https://www.arabidopsis.org/) were used to identify orthologs in the *B. napus* genome (Genbank accession number PRJNA237736) using BLAST (NCBI) and phylogenetic analysis was carried out using MUSCLE v3 [35] and MEGA v6 [36]. The phylogenetic tree was constructed using maximum-likelihood, the James–Taylor–Thorthon model and a bootstrap value of 1000. Sequences used in the analysis are provided in supplementary Table S3.

### Statistical analysis

The data were analyzed using the R Statistical Package [37]. Test and control groups, were compared using a two-sided Student’s t-test, while a one-way ANOVA followed by Tukey’s HSD post hoc test was used to compare the mean of multiple groups.

## Results

### *SSPbP53 is conserved among* P. brassicae *pathotypes*

**Figure 1.**
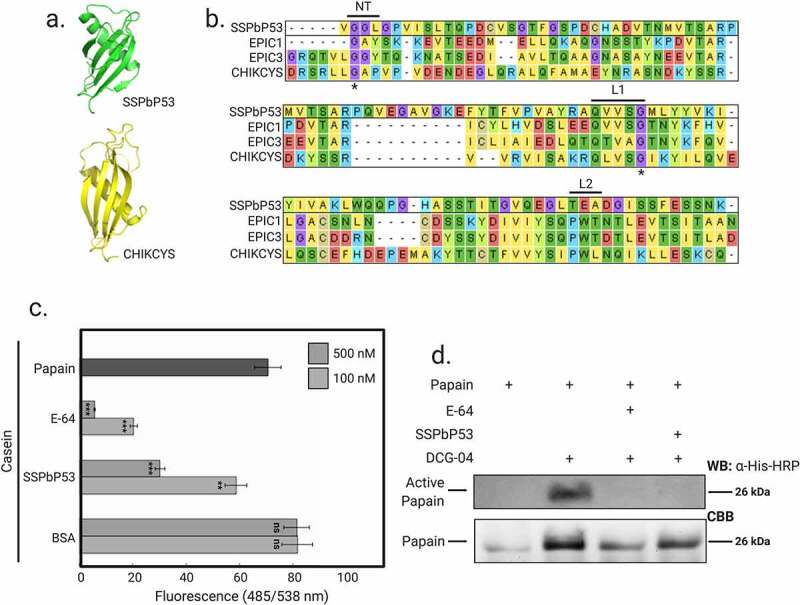
SSPbP53 is a putative cysteine protease inhibitor. **A**. Comparison of the 3D structure of SSPbP53 with the extracellular protease inhibitor with cystatin-like domain CHIKCYS. **B**. Sequence alignment of SSPbP53 and four other cysteine protease inhibitors showing the putative active site of the cystatins, including the N-terminal trunk (NT) and binding loops 1 and 2 (L1 and L2). Asterisks (*) indicate conserved amino acids in NT and L1. **C**. Proteolytic activity of papain, measured by digestion of a fluorescent casein substrate, is inhibited by E-64 (positive control) and purified His-SSPbP53 protein, but not BSA (negative control). The proteolytic activity of papain without inhibitors was also measured and included in the analysis. Fluorescence was measured at 485/538 nm excitation/emission. Mean ± standard deviation (n = 3). Statistically significant differences based on the two-tailed Student’s t-test are indicated by asterisks (*) where *p* < 0.01 is represented by (**), *p* < 0.001 by (***). ns – no significant difference. **D**. Activity-based protein profiling (ABPP) showing inhibition of papain by SSPbP53, preventing the subsequent binding of DCG-04 to papain

Among a number of SSPbPs, SSPbP53 was previously identified by our group as a cystatin-like protein [24]. SSPbP53 shares a common tertiary structure, an alpha helix lying on top of an anti-parallel beta sheet, with that of the extracellular protease inhibitor with cystatin-like domain (EPIC) proteins (Figure 1a), containing many of the signature sequences of cystatin-like protease inhibitors, including the conserved Gly residue in the N-terminal region and the highly conserved Gln-Xaa-Val-Xaa-Gly motif in the first binding loop (with Xaa represented by Val and Ser, respectively, in both SSPbP53 and EPIC1). However, a conserved Trp in the second binding loop [38] is not present in SSPbP53 (Figure 1b).

*SSPbP53* (PBRA_008207) was amplified from the Canadian *P. brassicae* pathotypes Pbc2, Pbc3, Pbc5, Pbc6, and Pbc8 and the nucleotide sequences of the resulting 500 bp amplicons were identical among all five pathotypes. The corresponding sequences, retrieved from the *P. brassicae* European pathotype e3 (GCA_001049375) and eH (GCA_003833335) genomes and the Chinese pathotype ZJ1 (GCA_002093825), also showed 100% nucleotide identity, resulting in a 100% shared amino acid sequence for SSPbP53 in all tested *P. brassicae* pathotypes.

### SSPbP53 inhibits plant papain-like cysteine proteases

To study SSPbP53 interactions with plant papain-like cysteine proteases (PLCPs), SSPbP53, minus the signal peptide and with an N-terminal His tag, was expressed in *E. coli* (Supplementary Fig. S1). SSPbP53 activity was first assessed using fluorescein-labeled casein as substrate, papain as the model PLCP and the chemical cysteine protease inhibitor E-64 as previously described [18,19,33]. SSPbP53 inhibited casein degradation by papain (Figure 1c). Inhibition of papain activity was dose-dependent with a > 50% reduction of activity achieved with 500 nM purified SSPbP53, comparable to that of 100 nM E-64 (Figure 1c, Supplementary Table S4). These concentrations for SSPbP53 and E-64 were used for all further analyses.

Using an activity-based protein profiling (ABPP) assay, together with E-64 and DCG-04, a biotinylated derivative of E-64, we further analyzed SSPbP53 activity [39]. The assay is based on the ability of E-64 or SSPbP53 to bind to papain or PCLPs, thereby blocking further binding of DCG-04 to the papain/PLCPs. Streptavidin beads were used to bind the biotin tag on DCG-04 and thereby measure the binding ability of SSPbP53 to papain/PCLPs, as indicated by a reduced streptavidin-horseradish peroxidase (HRP) signal on subsequent western blots. The pre-incubation of papain with either SSPbP53 or E-64 completely blocked any subsequent binding of DCG-04 to the papain (Figure 1d). These results show SSPbP53 to be a functional inhibitor of the model cysteine protease, papain.

### SSPbP53 localizes to the apoplast and inhibits cruciferous PLCP activity

**Figure 2.**
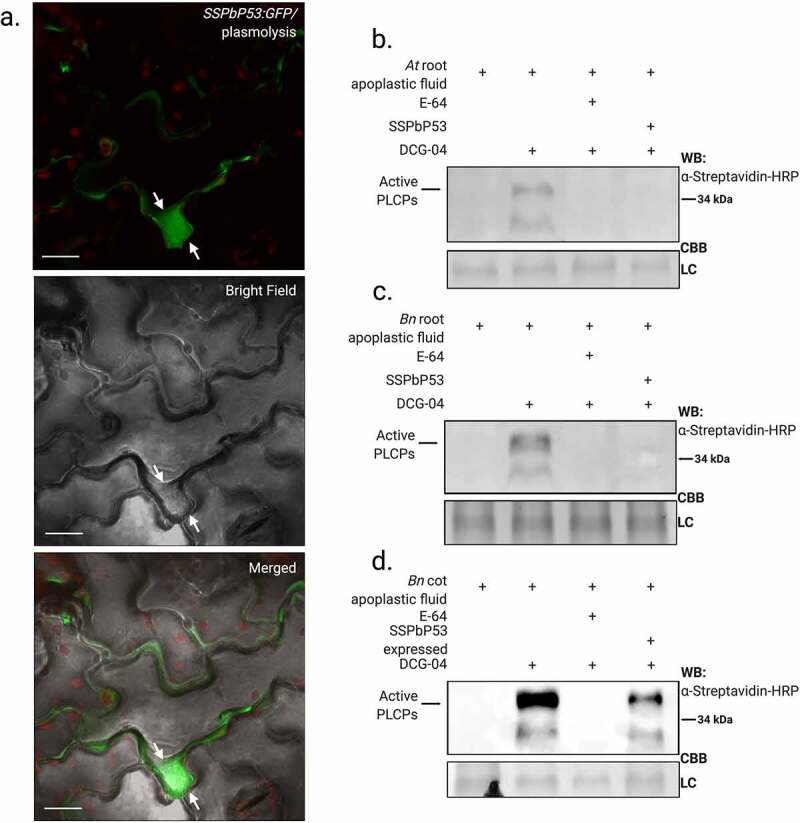
SSPbP53 is an apoplastic protein and inhibits apoplastic cruciferous PLCPs. **A**. Confocal images showing SSPbP53-GFP localized to the apoplast of rapeseed cotyledons, after salt-induced plasmolysis (Scale bars, 20 μM). Arrows indicate the retraction of the membrane from the cell wall. **B**. His-SSPbP53 inhibits Arabidopsis apoplastic PLCPs. Apoplastic proteins from Arabidopsis Col-0 roots were labeled via ABPP in the presence of purified His-SSPbP53. WB, western blot; CBB, coomassie brilliant blue; LC, Loading control. **C**. His-SSPbP53 inhibits apoplastic PLCPs in rapeseed roots. **D**. His-SSPbP53 transiently expressed in rapeseed cotyledons inhibits apoplastic PLCPs, *A. tumefaciens* containing empty vector (EV) was used as a negative control, together with non-treated cotyledons (Full figure in Supplementary Fig. S5)

ApoplastP [40] analysis predicted (P = 0.82) that SSPbP53 is an apoplastic protein. To confirm this prediction, *SSPbP53:GFP* was expressed in *B. napus* cotyledons (Supplementary Fig S2a). *SSPbP53-GFP* transcript was confirmed through RT-PCR and SSPbP53-GFP expression was confirmed through western blot using anti GFP-HRP (Supplementary Fig S2c-d). SSPbP53-GFP localized in the apoplastic space, with clear visualization of the cell wall dividing neighboring plant cells. Plasmolization of *B. napus* cotyledons further confirmed this apoplastic localization for SSPbP53-GFP (Figure 2e), when compared to SSPbP22-GFP, a previously characterized nuclear and cytoplasmic kinase [24], that remains in the cytoplasm and nucleus after plasmolysis (Supplementary Fig. S3). Similar apoplastic localization was observed for SSPbP53-GFP in cotyledons of *N. benthamiana* (Supplementary Fig S2b-e).

**Figure 3.**
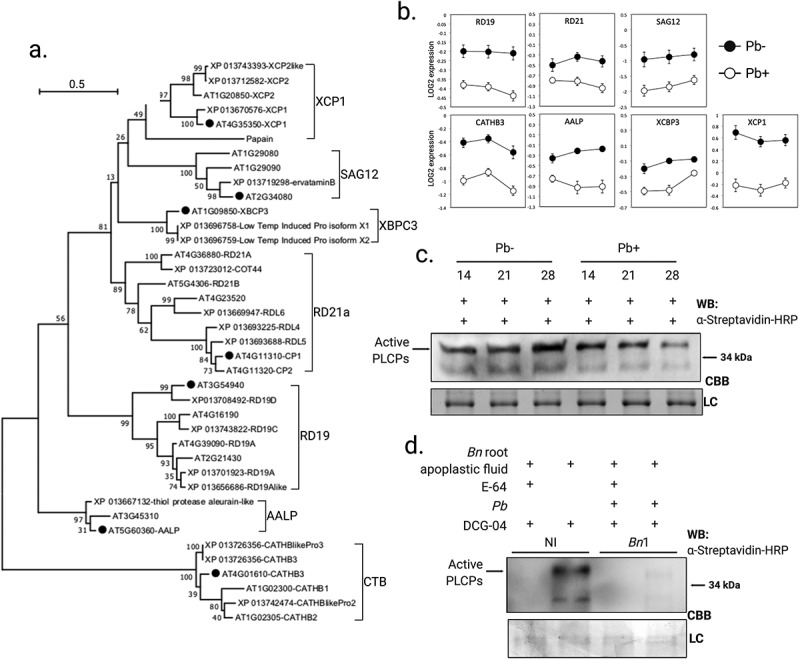
Levels of Arabidopsis and rapeseed PLCPs are reduced during *P. brassicae* infection. **A**. Phylogenetic tree of Arabidopsis and rapeseed orthologous PLCPs and subfamily classification. **B**. qRT-PCR amplification of Arabidopsis PLCPs, for which there are orthologs in the rapeseed genome, at 14, 21 and 24 dpi with *P. brassicae* (Pb+) or mock-inoculated (Pb-). Results are LOG2 expression relative to *P. brassicae ELONGATION FACTOR-LIKE* gene (*PbEF1*), normalized to expression in uninfected plants, and are the average of three biological replicates ±SE. **C**. Reduction of active apoplastic PLCPs in Pb+ Arabidopsis roots at 14, 21 and 24 dpi relative to Pb-. **D**. *P. brassicae* infection resulted in decreased availability of active apoplastic PLCPs in rapeseed roots at 21 dpi. NI, non-infected negative control

ABPP assays were carried out on root apoplastic fluid from Arabidopsis, *B. napus*, wild mustard (*Brassica kaber*), broccoli (*Brassica oleracea* var. italica), cabbage (*Brassica oleracea* var. capitate), and arugula (*Eruca vesicaria*). SSPbP53 almost completely inhibited biotinylation of PLCPs in all cruciferous root apoplastic fluids (Figure 3b-c, Supplementary Fig. S4). An ABPP assay of apoplastic fluid from *B. napus* cotyledons transiently expressing SSPbP53 without a fluorescence tag, also showed a reduced level of active apoplastic PLCPs (Figure 3d, Supplementary Fig. S5a). *SSPbP53* transcript, in agroinfiltrated *B. napus* cotyledons and 21 dpi *B. napus* roots, was confirmed through RT-PCR (Supplementary Fig. S5b). SSPbP53 did not inhibit *N. benthamiana* PLCPs (Supplementary Fig. S6), suggesting crucifer specificity for this *P. brassicae* effector.

### *Expression of PLCPs is down regulated in roots infected by* P. brassicae

The Arabidopsis genome encodes 32 PLCPs classified into eight main groups [41], of which, only seven orthologous groups are found in the *B. napus* genome (Genbank accession number PRJNA237736) (Figure 3a). To examine the expression of Arabidopsis PLCPs in *P. brassicae* infected roots a member from each of the seven orthologous groups was selected and transcript levels determined at 14, 21, and 28 dpi. Transcript levels for all investigated PLCPs were decreased in *P. brassicae* infected plants in comparison with mock-inoculated plants (Figure 3b). The down regulation was significant for *ALEURAIN-LIKE PROTEASE* (*AtAALP), CATHEPSIN B-LIKE PROTEASE 3* (*At*C*ATHB3*), *XYLEM BARK CYSTEINE PEPTIDASE 3* (*AtXBCP3*), and *XYLEM CYSTEINE PEPTIDASE 1* (*AtXCP1*) in *P. brassicae* infected plants (Figure 3b). Furthermore, active apoplastic PLCPs were also reduced in the roots of *P. brassica* infected (Pb+) Arabidopsis plants when compared to mock inoculated (Pb-) plants (Figure 3c). ABPPs were carried out on the apoplastic fluids from *B. napus*, arugula, broccoli, cabbage, and wild mustard roots at 21 dpi with *P. brassicae*, when *SSPbP53* transcript was highly expressed. Active apoplastic PLCPs were lower in *P. brassicae* infected cruciferous plants, when compared to non-infected (NI) plants (Figure 3d, Supplementary Fig. S7), suggesting that *P. brassicae* infection decreased total apoplastic PLCPs.

### *SSPbP53 interacts with xylem-associated PLCPs and the interaction contributes to plant host susceptibility to* P. brassicae

**Figure 4.**
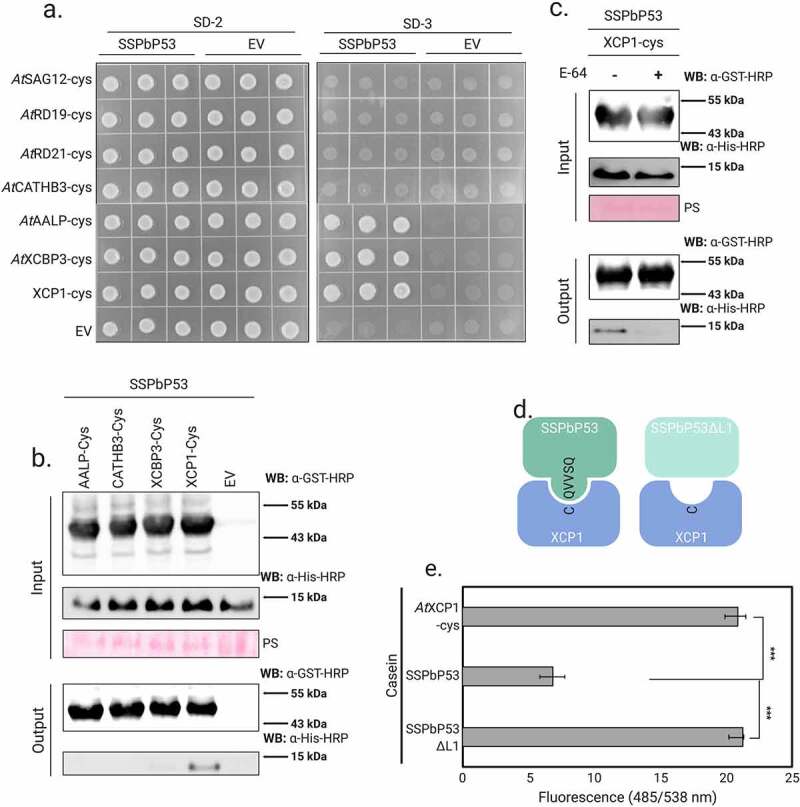
SSPbP53 interacts with xylem PLCPs. **A**. Yeast-two-hybrid (Y2H) assays using the clubroot pathogen SSPbP53 as the bait and Arabidopsis PLCP-cys domains representing different subfamilies as the prey. Growth of yeast cells on SD-3 selective media represents protein–protein interaction, while growth of the same cells on SD-2 media confirms yeast transformation. Yeast transformed with the empty vectors served as negative controls. **B**. *In vitro* pull-down assay using GST-*At*PLCP-cys to immunoprecipitate His-SSPbP53 protein. Input and output (immunoprecipitated proteins) were detected by western blotting (WB) using anti-GST and anti-His antibodies. *E. coli* transformed with empty GST-vector was used as negative control. Immunoprecipitated fraction was visualized through Ponceau stain (PS). **C**. Pre-incubation of GST-XCP1-cys with E-64 inhibits the interaction with His-SSPbP53. Input and output (immunoprecipitated proteins) were visualized by western blotting (WB) using anti-GST and anti-His antibodies. Immunoprecipitated fraction was visualized through Ponceau stain (PS). **D**. Schematic representation of SSPbP53 and SSPbP53^ΔL1^ interaction with XCP1. **E**. Proteolytic activity of His-XCP1-cys, measured by digestion of a fluorescent casein substrate, is inhibited by purified His-SSPbP53 protein, but not by SSPbP53^ΔL1^. Fluorescence was measured at 485/538 nm excitation/emission. Mean ± standard deviation (n = 3). Statistically significant differences based on the two-tailed Student’s t-test are indicated by asterisks (*) where *p* < 0.001 is represented by (***)

To determine if one or more of these Arabidopsis PLCPs can interact with SSPbP53, a pair-wise Y2H analysis was performed. The results showed that the bait yeast expressing SSPbP53 co transformed with *At*AALP, *At*XBCP3 and *At*XCP1 cysteine protease domains (*At*PLCP-cys) grew on SD-3 selective media (Figure 4a), while empty vector or the bait yeast only transformed with SSPbP53 was not able to grow. To confirm the results obtained through Y2H analysis, *in vitro* pull-down assays were carried out now for four of the initial PLCPs candidates evaluated. Each of *At*AALP-cys, *At*CATHB3-cys, *At*XBCP3-cys and *At*XCP1-cys cysteine protease domains with a GST tag at the N-terminus, was expressed in *E. coli*. The recombinant GST-PLCP-cys(s) were incubated separately with SSPbP53 and immunoprecipitated using glutathione agarose. SSPbP53 co-precipitated with *At*XCP1-cys and to a lesser degree *At*XBCP3-cys (Figure 4b). The interaction with *At*AALP-cys was not confirmed through pull-down, a more restrictive method than Y2H. Pre-incubation of *At*XCP1-cys with E-64 successfully prevented the *At*XCP1-SSPbP53 interaction (Figure 4c). The ability of SSPbP53 to inhibit *At*XCP1 activity and the importance of the loop 1 QVVAQ sequence (Figure 1b, Figure 4d), previously identified as essential for activity in cysteine proteases [42], to this interaction was assessed using fluorescein-labeled casein as substrate, SSPbP53^ΔL1^ (SSPbP53 mutant lacking the QVVAQ loop 1 sequence – Supplementary Fig. S8) and recombinant His-*At*XCP1-cys. SSPbP53 substantially inhibited casein degradation by *At*XCP1 whereas SSPbP53^ΔL1^ had no inhibitory effect on *At*XCP1 activity (Figure 4e, Supplementary Table S5), confirming the role of loop 1 in the inhibitory activity of SSPbP53.

**Figure 5.**
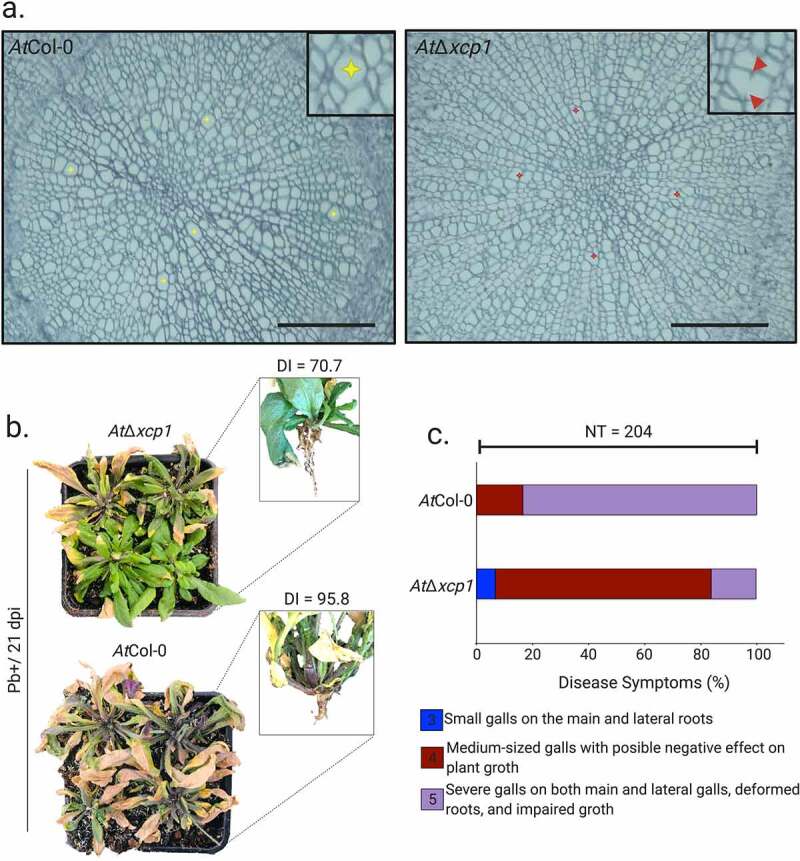
SSPbP53-*At*XCP1 interaction is required for Arabidopsis immunity. **A**. Light microscopy images of Pb+ *At*Δ*xcp1* and *At*Col-0 hypocotyl transverse sections at 21 dpi. Right upper corner amplification of expanded vessels indicated by yellow stars for *At*Col-0 and red arrowheads for *At*Δ*xcp1* (Scale bar – 50 μm). The observations presented are representative of 10 independent plants. **B**. Representation of the above and below ground of Pb+ *At*Δ*xcp1* mutant and *At*Col-0 at 21 dpi. **C**. Disease index for each group and graphic representation of the percentage of plants in each symptomatology for both groups, where NT is the total number of plants analyzed

To explore the *in planta* role of the *At*XCP1-SSPbP53 interaction, the clubroot disease index [24] was scored in the Arabidopsis *xcp1* null mutant (*At*Δ*xcp1*) at 21 dpi when, in infected *At*Col-0 plants, SSPbP53 is highly expressed and *At*XCP1is down regulated. Although XCP1 has been identified as involved in xylem maturation, *At*Δ*xcp1*mutants do not show abnormal vessel anatomy when compared with *At*Col-0 [43]. Transverse sections of the hypocotyl region of *AtΔxcp1* mutant and *At*Col-0 plants during secondary infection with *P. brassicae* did not show any major anatomical differences between the two sets of plant roots (Figure 5a). The only observed difference was that the beginning of the expansive phase [44] of the secondary infection was more evident in *At*Col-0 than in *AtΔxcp1* (Figure 5a). In *At*Δ*xcp1*, the cell wall destruction of neighbor cells that is characteristic of the expansion [24], was delayed compared to *At*Col-0 (Figure 5a). This was not observed in *At*Δ*xcp1* mutants Pb- (Supplementary Fig. S9a). A detailed response time course should be performed to confirm these observations.

Infected *At*Δ*xcp1* plants showed reduced susceptibility to *P. brassicae* infection and/or development of symptoms when compared to infected *At*Col-0 plants (Figure 5b-c, Supplementary Table S6). Furthermore, a lower number of resting spores was produced in *P. brassicae* infected *At*Δ*xcp1* plants compared to infected *At*Col-0 plants (Supplementary Fig. S9b, Supplementary Table S7), suggesting that a successful secondary infection and subsequent production of resting spores requires the inhibition of *At*XCP1 activity by SSPbP53. Null mutants of *At*Δ*rd19* and *At*Δ*rd21* did not show a decreased susceptibility to *P. brassicae* (Supplementary Fig. S9c-d, Supplementary Table S6), indicating that the *At*XCP1-SSPbP53 interaction is a specific requirement for *P. brassicae* pathogenicity.

## Discussion

Plant proteolytic enzymes are important players in the arms race between plants and their pathogens. Here, we report on the role of the *P. brassicae* effector SSPbP53 as a cysteine protease, specifically XCP1, inhibitor. We have shown that SSPbP53 is an apoplastic cystatin-like protein that inhibits the enzymatic activity of the Arabidopsis PLCP, *At*XCP1 (Figures 2 – 4). The results obtained from pull down experiments demonstrate that SSPbP53 interacts with two closely related xylem PLCPs, *At*XCPB3 and *At*XCP1 (Figure 4), but the interaction is clearly stronger with *At*XCP1. Xylem cysteine proteases like XCP1 and XCP2, members of the same PLCP group, play key roles in the pathogenicity of biotrophic pathogens [17,19]. In a maize-*U. maydis* compatible interaction (resulting in smut disease), the inhibition of maize XCP2 activity, that unhindered would result in plant cell death, by the endogenous cysteine protease inhibitor CC9, transcriptionally induced during epidermal penetration in the compatible interaction, is key to suppressing host immunity during infection [18]. *U. maydis* also secretes Pit2, a cystatin that mimics CC9 and targets XCP2 [19]. Pit2 is active in the apoplastic space where it inhibits host cysteine proteases and contributes to the suppression of host immunity [19]. In Arabidopsis, XCP2 activity is inhibited by the endogenous protein PRN2, that stabilizes XCP2 and prevents its autocatalytic degradation, even though PRN2 does not possess any known protease inhibitor domains such as those present in serpins and cystatins [45]. Arabidopsis *prn2, xcp2*, and *prn2-xcp2* null mutants are less susceptible than wild type to the vascular pathogen *Ralstonia solanacearum*, suggesting an essential role for XCP2 in plant susceptibility to this pathogen [45]. Interestingly, increased susceptibility to pathogens has also been reported when other PLCPs such as *At*RD19, a cysteine protease required for RRS1-R-mediated resistance [46], *At*RD21, an ortholog of the tomato immune protease C14 [47] and *Solanum lycopersicum* PIP1, an apoplastic PLCP [48] were knocked out.

A *P. brassicae* infection results in a reduction in active salicylic acid (SA) and its precursors in part due to the methylation of SA by PbBSMT [8]. We suggest that this reduced SA activity induces a down regulation of plant PLCP-encoding genes through a mechanism possibly similar to that recently described in maize [49]. SSPbP53 inhibits XCP1, XBCP3, and probably other PLCPs in the XCP1 group, thereby could be further contributing to the suppression of plant immunity. A very recent study has identified XCP1 as a caspase that proteolyzes Pathogenesis related protein 1, leading to the activation of systemic immunity [13]. SSPbP53 activity would be of particular importance during secondary infection, with its associated cell wall and vascular disruption [44,50] that would leave extracellular *P. brassicae* effectors susceptible to the protease activity of plant apoplastic PLCPs and cysteine proteases normally found in tertiary elements including XCP1. *At*Δ*xcp1* plants were less susceptible to *P. brassicae* than wild type plants or plants lacking other PLCPs suggesting that the XCP1-SSPbP53 interaction is important in cortical disruption and the development of the nutrient sink galls associated with clubroot disease. Under normal growth conditions, the *xcp1* mutation is complemented by increased expression of other PLCPs in the XCP1 group [43]. In Pb+ *At*Δ*xcp1* plants we observed incomplete breaking of the cell wall of neighbor cells, a normal process of the expansive phase of secondary infection (Figure 5). That SSPbP53 does not inhibit other PLCPs would allow a degree of normal root growth in infected *xcp1* plants. Something also very interesting in this study was the activity of SSPbP53 late during the secondary infection. Secondary infection is a vital infection stage for *P. brassicae* because it is when the pathogen initiates the expansion of cortical cells and the formation of galls, the nutrient rich niche required for completion of the life cycle and the formation of resting spores [44]. Other cysteine inhibitors with a similar function may act to protect the pathogen during primary infection, however, our study focused on secondary infection.

While it would be interesting to assess clubroot progression in *35S:XCP1* Arabidopsis lines, it has been reported that the over expression of *XCP1* is detrimental to plant growth [51]. It will also be of interest to investigate the response of Arabidopsis *35S:SSPbP53* lines to clubroot disease. However, we are confident that the results presented here are a good characterization of the effector and its role in clubroot progress.

This study is an important step toward understanding the mechanisms used by *P. brassicae* to induce galls during secondary infection and one of the very few studies characterizing an effector protein from *P. brassicae*, a plant pathogen that has proved to be a mystery for the plant pathology community. We hope this study will contribute to the generation of increased clubroot-resistant germplasm.

## Supplementary Material

Supplemental Material

## Data Availability

Data sharing is not applicable to this article as no new data were created or analyzed in this study.
