# Supplementary material for: A clubroot pathogen effector targets cruciferous cysteine proteases to suppress plant immunity
Source: Virulence. 2021 Sep 13;12(1):2327–40. doi: 10.1080/21505594.2021.1968684 (PMC8451464; doi:10.1080/21505594.2021.1968684)
Supplement: Supplemental Material [file KVIR_A_1968684_SM3048.zip › suppll/EPL-Supplementary Figure Legends-R1.docx]

**Fig. S1.** Recombinant His-SSPbP53. **A**. Schematic representation of the recombinant protein. **B**. Coomassie Brilliant Blue (CBB) gel of induced production of SSPbP53 (~15 kDa) by *E. coli* BL21DE3 post-induction with arabinose (Ind) and not induced (NoInd). **C**. Purification and concentration of His-SSPbP53 using Ni-NTA agarose.

**Fig. S2.** Confirmation that SSPbP53 is an apoplastic protein. **A-B**. SSPbP53-GFP was agroinfiltrated into rapeseed cotyledons (expression showed in Fig. 2 of main manuscript). Expression was confirmed through **C**. RT-PCR and **D**. western blot (WB) anti-GFP. CBB, coomassie brilliant blue; LC, Loading control. **E**. Confocal images showing SSPbP53-GFP localized to the apoplast of *Nicotiana benthamiana* leaves after salt-induced plasmolysis (Scale bars, 20 μM). Arrows indicate the retraction of the membrane from the cell wall.

**Fig. S3.** Intracellular localization control. **a.** Schematic representation of the SSPbP22 construct used as localization control. **b.** Fluorescence images showing SSPbP22-GFP localized to the nucleus (**n**) and cytoplasm (**c**) of *N. bethamiana* leaves. In the right panel we show how the localization of SSPbP22 remains intracellular when the cells were plasmolyzed with NaCl 5% showing membrane retraction from the cell wall (**cw**). (Scale bars, 20 μM).

**Fig. S4.** SSPbP53 inhibits cruciferous apoplastic PLCPs. Apoplastic protein extracts from. **A**. broccoli (*Brassica oleracea* Italica), **B**. cabbage (*B. oleracea* Capitata), **C**. wild mustard (*Brassica kaber*), and **D**. arugula (*Eruca vesicaria*) roots were labeled via ABPP in the presence of purified SSPbP53. WB, western blot; CBB, coomassie brilliant blue; LC, Loading control.

**Fig. S5.** SSPbP53 expression in cotyledons agroinfiltrated with *35S:SSPbP53* was confirmed through RT-PCR. **A**. SSPbP53-GFP transiently expressed in rapeseed cotyledons inhibits apoplastic PLCPs, *A. tumefaciens* containing empty vector (EV) was used as a negative control, together with non-treated (NT) cotyledons. Bn1 to Bn3 represents the three plants agroinfiltrated. **B**. RT-PCR confirming expression in agroinfiltrated rapeseed cotyledons using primers previously reported by Pérez-López et al. [22]

**Fig. S6.** SSPbP53 does not inhibit *Nicotiana benthamiana* PLCPs. Total protein extracts from *Nicotiana benthamiana* roots were labeled via ABPP in the presence of purified SSPbP53. WB, western blot; CBB, coomassie brilliant blue; LC, Loading control.

**Fig. S7.** Cruciferous PLCPs are reduced during *P. brassicae* infection at 21 dpi. Active PLCPs from apoplastic extracts of **A**. broccoli (*Brassica oleracea* Italica), **B**. wild mustard (*Eruca vesicaria*), **C**. arugula (*Brassica kaber*), **D**. cabbage (*B. oleracea* Capitata), and **E**. rapeseed (*Brassica napus*) roots were tagged using DCG-04. NT, non-treated negative control. WB, western blot; CBB, coomassie brilliant blue; LC, Loading control.

**Fig. S8.** Recombinant His-SSPbP53^ΔL1^ and His-XCP1-cys. **A**. Schematic representation of the sequence of the recombinant proteins with a thrombin cleavage site. **B**. Coomassie Brilliant Blue (CBB) gel of induced production of His-SSPbP53 (~15 kDa) as reference, His-SSPbP53^ΔL1^ (~14 kDa) and His-XCP1-cys (~27 kDa) by *E. coli* BL21DE3 cells after 2 h post-induction with arabinose.

**Fig. S9.** Comparison of Arabidopsis mutants and Col-0. **A**. Light microscopy images of Pb- *At*Δ*xcp1* hypocotyl at 21 days post mock-inoculation. (Scale bar, 50 μM). **B**. Spore production per plant of Pb+ Arabidopsis *At*Δ*xcp1* versus *At*Col-0 at 21 dpi. Statistically significant differences based on a one-way ANOVA followed by Tukey’s HSD post hoc test are indicated by asterisks where *p* < 0.05 is represented by (*). **C**. Representation of the above part of infected plants and graphic representation of the percentage of plants in each symptomatology for Pb+ *At*Δ*xcp1, At*Δ*rd19*, *At*Δ*rd21*, and *At*Col-0 at 21 dpi, where NT is the total number of plants analyzed. **D.** Arabidopsis Col-0 and *At*Δ*xcp1* Pb- at 21 days post mock-inoculation.
