# Supplementary material for: A clubroot pathogen effector targets cruciferous cysteine proteases to suppress plant immunity
Source: Virulence. 2021 Sep 13;12(1):2327–40. doi: 10.1080/21505594.2021.1968684 (PMC8451464; doi:10.1080/21505594.2021.1968684)
Supplement: Supplemental Material [file KVIR_A_1968684_SM3048.zip › suppll/EPL-Supplementary Tables-R1.docx]

**Table S1.** Primers used in this study.

| **Gene target (Genbank)** | **Primer name** | **Used for** | **Primer sequence (5’-3’)** |
| --- | --- | --- | --- |
| *SSPbP53 (CEP02396)* | SSPbP53attB1 | Gateway cloning into PDONZeo, PEarlyGate100 and 103, pDEST32 and pDEST17 | GGGGACAAGTTTGTACAAAAAAGCAGGCTTCATGGTGTTCCCGGGTGCCT |
|  | SSPbP53attB2 | Gateway cloning into PEarlyGate100, pDEST32 and pDEST17 | GGGGACCACTTTGTACAAGAAAGCTGGGTTTTATTATTTGTTGCTGCTCTCAA |
|  | SSPbP53attB2-no stop | Gateaway cloning into PEarlyGate103 | GGGGACCACTTTGTACAAGAAAGCTGGGTTGTGTTGCTGCTCTCAAGA |
|  | SSPbP53cDNAF | Amplification of SSPbP53 codifying sequence | ATGGTGTTCCCGGGTGCCT |
|  | SSPbP53cDNAR | Amplification of SSPbP53 codifying sequence | TTATTTGTTGCTGCTCTCAA |
|  | SSPbP53geneF | Amplification of SSPbP53 gene | ATGGTGTTCCCGGGTGC |
|  | SSPbP53geneR | Amplification of SSPbP53 gene | TTATTTGTTGCTGCTCTC |
| *XCP1* (AT4G35350) | AtXCP1F | RT-PCR | AAGAGACAGCCCTCAGCTAA |
|  | AtXCP1R | RT-PCR | CAGTGTTTCTCTTCATCCTA |
|  | AtXCP1attB1 | Gateway cloning into PDONZeo, pDEST22 and pDEST15 | GGGGACAAGTTTGTACAAAAAAGCAGGCTTCATGAAGAGACAGCCCTCAGCTAA |
|  | AtXCP1attB2 | Gateway cloning into PDONZeo, pDEST22 and pDEST15 | GGGGACCACTTTGTACAAGAAAGCTGGGTTTTACAGTGTTTCTCTTCATCCTA |
| *AALP* (AT5G60360) | AtAALPF | RT-PCR | AAGGTCACAGAAGCAGCT |
|  | AtAALPR | RT-PCR | CCAATCCGCTCCCCATGA |
|  | AtAALPattB1 | Gateway cloning into PDONZeo, pDEST22 and pDEST15 | GGGGACAAGTTTGTACAAAAAAGCAGGCTTCATGAAGGTCACAGAAGCAGCT |
|  | AtAALPattB2 | Gateway cloning into PDONZeo, pDEST22 and pDEST15 | GGGGACCACTTTGTACAAGAAAGCTGGGTTTTACCAATCCGCTCCCCATGA |
| *CATHB3* (AT4G01610) | AtCATHB3F | RT-PCR | GGCTTCTCTTGGCTTTTG |
|  | AtCATHB3R | RT-PCR | AGTTCCCCAACCAATAAGTT |
|  | AtCATHB3attB1 | Gateway cloning into PDONZeo, pDEST22 and pDEST15 | GGGGACAAGTTTGTACAAAAAAGCAGGCTTCATGGGCTTCTCTTGGCTTTTG |
|  | AtCATHB3attB2 | Gateway cloning into PDONZeo, pDEST22 and pDEST15 | GGGGACCACTTTGTACAAGAAAGCTGGGTTTTAAGTTCCCCAACCAATAAGTT |
| *XBCP3* (AT1G09850) | AtXBCP3F | RT-PCR | ATGGTTACTAATGTCAAAGA |
|  | AtXBCP3R | RT-PCR | GTGTTACGCTGCATGTGC |
|  | AtXBCP3attB1 | Gateway cloning into PDONZeo, pDEST22 and pDEST15 | GGGGACAAGTTTGTACAAAAAAGCAGGCTTCATGATGGTTACTAATGTCAAAGA |
|  | AtXBCP3attB2 | Gateway cloning into PDONZeo, pDEST22 and pDEST15 | GGGGACCACTTTGTACAAGAAAGCTGGGTTTTAGTGTTACGCTGCATGTGC |
| *SAG12* (AT5G45890) | AtSAG12F | RT-PCR | ACAAAGGCGAAGACGCTACT |
|  | AtSAG12R | RT-PCR | ATTCGCCGTATCCAATCGCA |
|  | AtSAG12attB1 | Gateway cloning into PDONZeo and pDEST22 | GGGGACAAGTTTGTACAAAAAAGCAGGCTTCATGCCGGTTTCTGTTGA |
|  | AtSAG12attB2 | Gateway cloning into PDONZeo and pDEST22 | GGGGACCACTTTGTACAAGAAAGCTGGGTTTTATCATATAGTTGGGTAAGA |
| *AtRD19* (AT4G39090) | AtRD19F | RT-PCR | GGCAAGACCTGCAAGCTAGA |
|  | AtRD19R | RT-PCR | TAAGACACCGTGGTTGAGCC |
|  | AtRD19attB1 | Gateway cloning into PDONZeo and pDEST22 | GGGGACAAGTTTGTACAAAAAAGCAGGCTTC ATGGAGCCGAGCCTCAG |
|  | AtRD19attB2 | Gateway cloning into PDONZeo and pDEST22 | GGGGACCACTTTGTACAAGAAAGCTGGGTTTTAAACGGCCCAGACTTGATT |
| *At*RD21 (AT1G47128) | *At*RD21F | RT-PCR | GGGTTTGACTCGTTTTGCGG |
|  | *At*RD21R | RT-PCR | GAAAACGCCCAACAACTCCC |
|  | *At*RD21attB1 | Gateway cloning into PDONZeo and pDEST22 | GGGGACAAGTTTGTACAAAAAAGCAGGCTTCATGTTAGTTATAGATTGGG |
|  | *At*RD21attB2 | Gateway cloning into PDONZeo and pDEST22 | GGGGACCACTTTGTACAAGAAAGCTGGGTTTTATTCCGTATCCAACCGCCA |

**Table S2.** Plasmids generated in this study.

| **Plasmid** | **Backbone vector** | **Insert DNA fragments** | **Cloning strategy** |
| --- | --- | --- | --- |
| pDONZeo_SSPbP53 | pDONR/Zeo | Open reading frame of SSPbP53 lacking signal peptide | Gateway system |
| pDEST17_SSPbP53 | pDEST17 | Open reading frame of SSPbP53 lacking signal peptide | Gateway system |
| pEG100_SSPbP53 | pEarlyGate100 | Open reading frame of SSPbP53 lacking signal peptide | Gateway system |
| pEG103_SSPbP53 | pEarlyGate103 | Open reading frame of SSPbP53 lacking signal peptide | Gateway system |
| pDEST32_SSPbP53 | pDEST32 | Open reading frame of SSPbP53 lacking signal peptide | Gateway system |
| pET-14b_SSPbP53ΔL1 | pET-14b | Open reading frame of SSPbP53 lacking signal peptide and loop 1 | Restriction enzymes (5' XhoI and 3' BamHI) |
| pDEST32_ SSPbP53 | pDEST32 | Open reading frame of SSPbP53 lacking signal peptide | Gateway system |
| pDONZeo_AtAALP-cys | pDONR/Zeo | 594 bp fragment of AtAALP codifying Cysteine protease domain | Gateway system |
| pDEST15__AtAALP-cys | pDEST15 | 594 bp fragment of AtAALP codifying Cysteine protease domain | Gateway system |
| pDEST22__AtAALP-cys | pDEST22 | 594 bp fragment of AtAALP codifying Cysteine protease domain | Gateway system |
| pDONZeo_AtCATHB3-cys | pDONR/Zeo | 606 bp fragment of AtCATHB3 codifying Cysteine protease domain | Gateway system |
| pDEST15__AtCATHB3-cys | pDEST15 | 606 bp fragment of AtCATHB3 codifying Cysteine protease domain | Gateway system |
| pDEST22__AtCATHB3-cys | pDEST22 | 606 bp fragment of AtCATHB3 codifying Cysteine protease domain | Gateway system |
| pDONZeo_AtXBCP3-cys | pDONR/Zeo | 585 bp fragment of AtXBCP3 codifying Cysteine protease domain | Gateway system |
| pDEST15__AtXBCP3-cys | pDEST15 | 585 bp fragment of AtXBCP3 codifying Cysteine protease domain | Gateway system |
| pDEST22__AtXBCP3-cys | pDEST22 | 585 bp fragment of AtXBCP3 codifying Cysteine protease domain | Gateway system |
| pDONZeo_AtXCP1-cys | pDONR/Zeo | 609 bp fragment of AtXCP1 codifying Cysteine protease domain | Gateway system |
| pET-14b_AtXCP1-cys | pET-14b | 609 bp fragment of AtXCP1 codifying Cysteine protease domain | Restriction enzymes (5' XhoI and 3' BamHI) |
| pDEST15__AtXCP1-cys | pDEST15 | 609 bp fragment of AtXCP1 codifying Cysteine protease domain | Gateway system |
| pDEST22__AtXCP1-cys | pDEST22 | 609 bp fragment of AtXCP1 codifying Cysteine protease domain | Gateway system |
| pDONR/Zeo_ *At*SAG12-cys | pDONR/Zeo | 651 bp fragment of *At*SAG12 codifying Cysteine protease domain | Gateway system |
| pDEST22_ *At*SAG12-cys | pDEST22 | 651 bp fragment of *At*SAG12 codifying Cysteine protease domain | Gateway system |
| pDONR/Zeo_ *At*RD19-cys | pDONR/Zeo | 1276 bp fragment of *At*RD19 codifying Cysteine protease domain | Gateway system |
| pDEST2_ *At*RD19-cys | pDEST22 | 1276 bp fragment of *At*RD19 codifying Cysteine protease domain | Gateway system |
| pDONR/Zeo_ *At*RD21-cys | pDONR/Zeo | 645 bp fragment of *At*RD21 codifying Cysteine protease domain | Gateway system |
| pDEST22_ *At*RD21-cys | pDEST22 | 645 bp fragment of *At*RD21 codifying Cysteine protease domain | Gateway system |

**Table S3.** PLCPs sequences used in the phylogenetic analysis.

| **Gene *** | **Genbank Accession** |
| --- | --- |
| *AtCATHB1* | AT1G02300 |
| *AtCATHB2* | AT1G02305 |
| *AtXCP2* | AT1G20850 |
| At cysteine protease | AT1G29090 |
| *RD21A* | AT1G47128 |
| *At* papain family cysteine protease | AT2G21430 |
| *At* cysteine protease | AT3G45310 |
| *AtCATHB3* | AT4G01610 |
| *At* papain family cysteine protease | AT4G11320 |
| *At* papain family cysteine protease | AT4G16190 |
| At cysteine protease | AT4G23520 |
| *At*CP1 | AT4G36880 |
| *AtRD19A* | AT4G39090 |
| *AtRD21B* | AT5G4306 |
| Papain | NW_019013654 |
| BnCATHBlikePro2 | XP_013742474 |
| BnCATHBlikePro3 | XP_013726356 |
| AtXBCP3 | AT1G09850 |
| Bn Low Temp Induced Pro isoform X1 | XP_013696758 |
| Bn Low Temp Induced Pro isoform X2 | XP_013696759 |
| Bn XCP2like | XP_013743393 |
| Bn XCP2 | XP_013712582 |
| Bn XCP1 | XP_013670576 |
| At papain family cysteine protease | AT1G29080 |
| Bn ErvataminB | XP_013719298 |
| Bn RD19A | XP_013701923 |
| Bn RD19Alike | XP_013656686 |
| At cysteine protease | AT2G34080 |
| At cysteine protease | AT3G45310 |
| At papain family cysteine protease | AT3G54940 |
| Bn probable RD19D | XP_013708492 |
| Bn CATHB3 | XP_013726356 |
| BnRDL4 | XP_013693225 |
| AtCP2 | AT4G11320 |
| Bn RDL5 | XP_013693688 |
| At papain family cysteine protease | AT4G16190 |
| Bn RD19C | XP_013743822 |
| At cysteine protease | AT4G23520 |
| Bn RDL6 | XP_013669947 |
| AtXCP1 | AT4G35350 |
| BnXCP1 | XP_013670576 |
| BnCOT44 | XP_013723012 |
| AtAALP | AT5G60360 |
| Bn thiol protease aleurain-like | XP_013667132 |
| * **At** from Arabidopsis and **Bn** from *Brassica napus* |  |

**Table S4.** Raw data for *in vitro* inhibition fluorescence results at 485/538 nm (SSPbP53 inhibition assay).

| BSA 100 | BSA 500 | E64 100 | E64 500 | SSPbP53 100 | SSPbP53 500 | Papain | Casein |
| --- | --- | --- | --- | --- | --- | --- | --- |
| 89.61 | 83.46 | 29.07 | 8.2 | 63.55 | 32.57 | 78.05 | 4.74 |
| 84.93 | 80.56 | 23.33 | 8.06 | 61.86 | 37.8 | 73.1 | 5.94 |
| 88.04 | 84.64 | 27.31 | 8.54 | 61.32 | 32.32 | 73.76 | 5.88 |
| 82.29 | 88.5 | 24.31 | 10.5 | 67.73 | 34.7 | 74.41 | 5.17 |
| 83.68 | 85.58 | 24.52 | 12.94 | 63.67 | 33.62 | 73.17 | 5.92 |
| 88.58 | 85.28 | 25.39 | 12.93 | 63.1 | 32.19 | 76.41 | 5.37 |
| 87.33 | 83.57 | 21.67 | 9.22 | 64.24 | 31.04 | 72.59 | 2.54 |
| 87.38 | 89 | 21.33 | 11.37 | 65.22 | 35.5 | 75.79 | 5.26 |
| 82.79 | 86.89 | 22.47 | 10.51 | 64.76 | 32.07 | 72.1 | 2.89 |
| BSA 100 | BSA 500 | E64 100 | E64 500 | SSPbP53 100 | SSPbP53 500 | Papain | Casein |
| 84.25 | 86.81 | 29.38 | 8.4 | 60.48 | 33.03 | 72.16 | 5.16 |
| 88.88 | 81.58 | 29.56 | 7.8 | 60.18 | 36.88 | 78.1 | 3.96 |
| 84.74 | 81.84 | 28.46 | 8.25 | 60.14 | 35.3 | 75.36 | 3.7 |
| 84.83 | 88.76 | 22.13 | 12.15 | 64.39 | 33.47 | 73.99 | 4.54 |
| 85.05 | 85.67 | 23.29 | 12.57 | 63.67 | 32.72 | 70.66 | 5.62 |
| 84.8 | 85.55 | 26.8 | 10.14 | 66.87 | 34.83 | 79.39 | 4.51 |
| 85.45 | 86.66 | 24.69 | 11.02 | 62.61 | 38.64 | 77.57 | 5.51 |
| 84.69 | 89.08 | 26.28 | 9.42 | 64.8 | 35.89 | 77.21 | 5.95 |
| 85.35 | 87.05 | 20.62 | 13.13 | 64.15 | 34.82 | 73.37 | 6.93 |
| BSA 100 | BSA 500 | E64 100 | E64 500 | SSPbP53 100 | SSPbP53 500 | Papain | Casein |
| 85.11 | 89.86 | 22.48 | 7.07 | 62.88 | 36.22 | 77.61 | 5.64 |
| 85.87 | 87.2 | 25.23 | 8.82 | 59.15 | 32.94 | 75.05 | 5.76 |
| 87.13 | 80.29 | 28.51 | 7.33 | 57.49 | 35.27 | 76.78 | 5.81 |
| 86.98 | 85.35 | 23.9 | 12.46 | 60.39 | 36.76 | 70.18 | 5.48 |
| 88.6 | 85.65 | 25.2 | 12.49 | 64.17 | 36.76 | 79.39 | 5.35 |
| 85.74 | 85.72 | 30.55 | 11.34 | 64 | 31.92 | 78.08 | 5.97 |
| 86.82 | 86.9 | 23.14 | 10.42 | 61.6 | 37.61 | 74.99 | 2.33 |
| 86.65 | 88.46 | 21.22 | 10.37 | 65.24 | 36.76 | 74.78 | 5.45 |
| 85.79 | 86.23 | 21.39 | 11.84 | 64.73 | 34.67 | 76.77 | 3.25 |

**Table S5.** Raw data for *in vitro* inhibition fluorescence results at 485/538 nm (XCP1 activity and SSPbP53^ΔL1^ inhibition assays).

| XCP1 | XCP1+SSPbP53 | XCP1+SSPbP53ΔL1 |
| --- | --- | --- |
| 20.404 | 6.108 | 21.465 |
| 21.204 | 5.952 | 21.398 |
| 21.24 | 6.164 | 21.607 |
| 20.666 | 6.404 | 21.687 |
| 21.185 | 6.299 | 21.687 |
| 21.884 | 6.385 | 21.462 |
| XCP1 | XCP1+SSPbP53 | XCP1+SSPbP53ΔL1 |
| 21.126 | 6.281 | 20.524 |
| 21.448 | 6.707 | 19.829 |
| 21.445 | 5.825 | 20.294 |
| 21.62 | 7.109 | 20.502 |
| 21.456 | 7.064 | 20.619 |
| 21.822 | 7.235 | 20.705 |
| XCP1 | XCP1+SSPbP53 | XCP1+SSPbP53ΔL1 |
| 19.2 | 7.164 | 20.653 |
| 21.677 | 7.865 | 20.298 |
| 22.294 | 7.21 | 20.373 |
| 19.605 | 8 | 20.78 |
| 21.879 | 8.713 | 21.221 |
| 22.075 | 7.163 | 20.494 |

**Table S6.** Raw data for infection assay of *At*Δ*xcp1, At*Δ*rd19*, *At*Δ*rd21* and *At*Col-0 at 21 dpi.

| **R1** | Disease level | | | | | Total | DI/Replicate |
| --- | --- | --- | --- | --- | --- | --- | --- |
|  | **0** | **1** | **2** | **3** | **4** |  |  |
| *At*Δ*xcp*1 | 0 | 0 | 6 | 52 | 10 | 68 | 76.5 |
| AtCol-0 | 0 | 0 | 0 | 7 | 61 | 68 | 97.4 |
| *At*Δ*rd*19 | 0 | 0 | 1 | 12 | 55 | 68 | 94.8 |
| *At*Δ*rd*21 | 0 | 0 | 0 | 17 | 51 | 68 | 93.75 |
| **R2** |  |  |  |  |  | 204 |  |
|  | **0** | **1** | **2** | **3** | **4** |  |  |
| *At*Δ*xcp*1 | 0 | 0 | 3 | 48 | 17 | 68 | 80.1 |
| AtCol-0 | 0 | 0 | 0 | 12 | 56 | 68 | 95.6 |
| *At*Δ*rd*19 | 0 | 0 | 0 | 10 | 58 | 68 | 96.3 |
| *At*Δ*rd*21 | 0 | 0 | 2 | 13 | 53 | 68 | 93.75 |
| **R3** |  |  |  |  |  | 204 |  |
|  | **0** | **1** | **2** | **3** | **4** |  |  |
| *At*Δ*xcp*1 | 0 | 0 | 5 | 57 | 6 | 68 | 75.4 |
| AtCol-0 | 0 | 0 | 0 | 15 | 53 | 68 | 94.5 |
| *At*Δ*rd*19 | 0 | 0 | 0 | 16 | 52 | 68 | 94.1 |
| *At*Δ*rd*21 | 0 | 0 | 1 | 14 | 53 | 68 | 94.1 |
|  |  |  |  |  |  | 204 |  |
|  | **0** | **1** | **2** | **3** | **4** |  | DI total |
| *At*Δ*xcp*1 | 0 | 0 | 14 | 157 | 33 | 204 | 77.3 |
| AtCol-0 | 0 | 0 | 0 | 34 | 170 | 204 | 95.8 |
| *At*Δ*rd*19 | 0 | 0 | 1 | 38 | 165 | 204 | 95 |
| *At*Δ*rd*21 | 0 | 0 | 3 | 44 | 157 | 204 | 93.8 |
|  | % | | | |  |  |  |
| Disease Level | *At*Δ*xcp1* | AtCol-0 | *At*Δ*rd19* | *At*Δ*rd21* |  |  |  |
| 0 | 0 | 0 | 0 | 0 |  |  |  |
| 1 | 0 | 0 | 0 | 0 |  |  |  |
| 2 | 6.8 | 0 | 0.49 | 1.47 |  |  |  |
| 3 | 76.9 | 16.6 | 18.6 | 21.5 |  |  |  |
| 4 | 16.1 | 83.3 | 80.8 | 76.9 |  |  |  |

**Table S7.** Raw data for spore production by infected *At*Δ*xcp1* and *At*Col-0 at 21 dpi.

| ***At*Δ*xcp1*** |  |  |  | **Spores in 0.5 g dry weight count** | **Spores in 1 g dry weight count** |
| --- | --- | --- | --- | --- | --- |
| 65 | 82 | 73 | 67 | 71.4 | 142.9 |
| 81 | 71 | 80 | 87 | 79.5 | 159.0 |
| 60 | 65 | 63 | 72 | 64.8 | 129.7 |
| 78 | 73 | 69 | 87 | 76.4 | 152.9 |
| 95 | 91 | 80 | 105 | 92.3 | 184.6 |
| 83 | 88 | 81 | 83 | 83.7 | 167.4 |
| 73 | 75 | 70 | 76 | 73.4 | 146.9 |
| 67 | 60 | 68 | 70 | 66.1 | 132.2 |
| 70 | 76 | 69 | 80 | 73.6 | 147.2 |
| 85 | 82 | 81 | 79 | 81.7 | 163.4 |
| 70 | 65 | 68 | 62 | 66.1 | 132.3 |
| 68 | 75 | 81 | 76 | 74.8 | 149.7 |
| 91 | 93 | 85 | 82 | 87.6 | 175.2 |
| 79 | 78 | 66 | 72 | 73.5 | 147.1 |
| 82 | 83 | 65 | 61 | 72.0 | 144.1 |
| 73 | 75 | 81 | 78 | 76.6 | 153.3 |
| 68 | 72 | 80 | 67 | 71.5 | 143.1 |
| 81 | 86 | 82 | 80 | 82.2 | 164.4 |
| 63 | 66 | 69 | 61 | 64.6 | 129.3 |
| 67 | 68 | 72 | 70 | 69.2 | 138.4 |
| 81 | 75 | 73 | 91 | 79.7 | 159.4 |
| 89 | 90 | 79 | 82 | 84.8 | 169.7 |
| 62 | 60 | 58 | 61 | 60.2 | 120.4 |
| 73 | 70 | 69 | 62 | 68.3 | 136.7 |
| 70 | 68 | 60 | 59 | 64.0 | 128.1 |
| 72 | 85 | 87 | 80 | 80.7 | 161.5 |
| 87 | 78 | 65 | 70 | 74.5 | 149.0 |
| 76 | 68 | 72 | 67 | 70.6 | 141.3 |
| 79 | 83 | 80 | 76 | 79.4 | 158.9 |
| 68 | 72 | 75 | 67 | 70.4 | 140.8 |
| 82 | 80 | 86 | 87 | 83.7 | 167.4 |
| 71 | 77 | 82 | 73 | 75.6 | 151.2 |
| 66 | 62 | 68 | 60 | 63.9 | 127.8 |
| 59 | 62 | 63 | 82 | 65.9 | 131.8 |
| 68 | 61 | 60 | 62 | 62.6 | 125.3 |
| 90 | 88 | 79 | 85 | 85.3 | 170.7 |
| 81 | 69 | 75 | 82 | 76.5 | 153.1 |
| 58 | 62 | 57 | 65 | 60.4 | 120.8 |
| 86 | 80 | 82 | 85 | 83.2 | 166.4 |
| 100 | 92 | 98 | 86 | 93.8 | 187.6 |
| 92 | 96 | 100 | 86 | 93.3 | 186.7 |
| 88 | 86 | 92 | 78 | 85.8 | 171.6 |
| 69 | 82 | 66 | 62 | 69.3 | 138.7 |
| 71 | 67 | 62 | 71 | 67.6 | 135.2 |
| 66 | 70 | 69 | 73 | 69.4 | 138.9 |
| 90 | 74 | 72 | 80 | 78.6 | 157.3 |
| 76 | 82 | 69 | 77 | 75.8 | 151.7 |
| 71 | 69 | 61 | 63 | 65.8 | 131.8 |
| 80 | 79 | 70 | 74 | 75.6 | 151.2 |
| 86 | 83 | 82 | 80 | 82.7 | 165.4 |
| 68 | 71 | 65 | 73 | 69.1 | 138.3 |

| **weight** | **Spores per**  **pot count** | **Spores per**  **plant count** | **Spores per plant**  **hemocytometer**  **standardized** |
| --- | --- | --- | --- |
| 68 | 9717.8 | 2429.4 | 24294621.3 |
| 68 | 10817.5 | 2704.3 | 27043759.7 |
| 68 | 8820.1 | 2205.0 | 22050240.4 |
| 68 | 10398.8 | 2599.7 | 25997127.7 |
| 68 | 12554.5 | 3138.6 | 31386290.3 |
| 68 | 11384.6 | 2846.1 | 28461637. |
| 68 | 9991.1 | 2497.8 | 24977723.6 |
| 68 | 8994.9 | 2248.7 | 22487292.4 |
| 68 | 10011.5 | 2502.9 | 25028890.2 |
| 68 | 11114.1 | 2778.5 | 27785303.1 |
| 68 | 9000.5 | 2250.1 | 22501264.1 |
| 68 | 10180.2 | 2545.0 | 25450487.9 |
| 68 | 11918.7 | 2979.7 | 29796701.9 |
| 68 | 10004.3 | 2501.1 | 25010849.8 |
| 68 | 9802.2 | 2450.5 | 24505451.0 |
| 68 | 10429.9 | 2607.4 | 26074732 |
| 68 | 9733.9 | 2433.5 | 24334950.4 |
| 68 | 11181.7 | 2795.4 | 27954436.6 |
| 68 | 8796.3 | 2199.1 | 21990989.4 |
| 68 | 9414.4 | 2353.6 | 23535990.5 |
| 68 | 10839.7 | 2709.9 | 27099205.03 |
| 68 | 11542.6 | 2885.6 | 28856741.0 |
| 68 | 8191.5 | 2047.8 | 20478781.3 |
| 68 | 9299.3 | 2324.8 | 23248467.8 |
| 68 | 8713.4 | 2178.3 | 21783691.1 |
| 68 | 10986.9 | 2746.7 | 27467496.7 |
| 68 | 10137.8 | 2534.4 | 25344587.6 |
| 68 | 9609.9 | 2402.4 | 24024887.8 |
| 68 | 10806.6 | 2701.6 | 27016625.8 |
| 68 | 9578.1 | 2394.5 | 23945465.5 |
| 68 | 11383.3 | 2845.8 | 28458322.9 |
| 68 | 10286.3 | 2571.6 | 25715819.2 |
| 68 | 8693.3 | 2173.3 | 21733409.5 |
| 68 | 8966.8 | 2241.7 | 22417049.0 |
| 68 | 8523.8 | 2130.9 | 21309552.9 |
| 68 | 11613.9 | 2903.5 | 29034992.4 |
| 68 | 10413.3 | 2603.3 | 26033419.9 |
| 68 | 8216.5 | 2054.1 | 20541440.2 |
| 68 | 11317.3 | 2829.3 | 28293340.1 |
| 68 | 12761.9 | 3190.4 | 31904871.9 |
| 68 | 12696.3 | 3174.1 | 31740831.1 |
| 68 | 11674.9 | 2918.7 | 29187359.3 |
| 68 | 9433.8 | 2358.4 | 23584602.4 |
| 68 | 9199.9 | 2299.9 | 22999834.6 |
| 68 | 9445.8 | 2361.4 | 23614698.1 |
| 68 | 10703.1 | 2675.7 | 26757965.8 |
| 68 | 10316.4 | 2579.1 | 25791151.4 |
| 68 | 8958.4 | 2239.6 | 22396150.2 |
| 68 | 10287.3 | 2571.8 | 25718203.3 |
| 68 | 11250.2 | 2812.5 | 28125419.7 |
| 68 | 9408.9 | 2352.2 | 23522300.1 |

| **AtCol-0** |  |  |  | **Spores in**  **0.5 g dry**  **weight count** | **Spores in**  **1 g dry**  **weight count** |
| --- | --- | --- | --- | --- | --- |
| 113 | 105 | 108 | 100 | 106.3 | 212.7 |
| 98 | 101 | 118 | 99 | 103.6 | 207.3 |
| 105 | 101 | 92 | 95 | 98.1 | 196.2 |
| 125 | 110 | 115 | 99 | 111.8 | 223.7 |
| 118 | 122 | 100 | 105 | 110.8 | 221.7 |
| 121 | 98 | 132 | 97 | 111.0 | 222.0 |
| 126 | 119 | 122 | 112 | 119.6 | 239.2 |
| 103 | 110 | 102 | 116 | 107.6 | 215.2 |
| 111 | 108 | 115 | 103 | 109.1 | 218.3 |
| 132 | 124 | 117 | 130 | 125.6 | 251.2 |
| 114 | 128 | 131 | 125 | 124.3 | 248.6 |
| 139 | 123 | 109 | 124 | 123.2 | 246.5 |
| 122 | 120 | 104 | 118 | 115.7 | 231.5 |
| 131 | 112 | 108 | 125 | 118.6 | 237.2 |
| 119 | 128 | 133 | 109 | 121.9 | 243.8 |
| 125 | 120 | 123 | 119 | 121.7 | 243.4 |
| 114 | 106 | 134 | 102 | 113.3 | 226.7 |
| 129 | 112 | 119 | 113 | 118.0 | 236.1 |
| 125 | 118 | 130 | 114 | 121.5 | 243.1 |
| 115 | 132 | 108 | 117 | 117.6 | 235.3 |
| 121 | 110 | 123 | 119 | 118.1 | 236.2 |
| 138 | 131 | 133 | 136 | 134.4 | 268.9 |
| 128 | 132 | 127 | 122 | 127.1 | 254.3 |
| 103 | 119 | 111 | 127 | 114.6 | 229.3 |
| 136 | 126 | 118 | 124 | 125.8 | 251.6 |
| 138 | 112 | 130 | 126 | 126.1 | 252.2 |
| 124 | 128 | 131 | 125 | 126.9 | 253.9 |
| 118 | 115 | 124 | 131 | 121.8 | 243.6 |
| 122 | 115 | 128 | 139 | 125.6 | 251.3 |
| 120 | 110 | 133 | 116 | 119.4 | 238.9 |
| 137 | 126 | 112 | 105 | 119.3 | 238.7 |
| 117 | 135 | 132 | 121 | 126.0 | 252.1 |
| 103 | 122 | 131 | 128 | 120.4 | 240.9 |
| 109 | 123 | 111 | 132 | 118.3 | 236.7 |
| 112 | 132 | 108 | 102 | 112.9 | 225.9 |
| 135 | 126 | 110 | 145 | 128.3 | 256.6 |
| 141 | 126 | 136 | 132 | 133.6 | 267.3 |
| 133 | 126 | 105 | 129 | 122.7 | 245.5 |
| 142 | 123 | 109 | 133 | 126.1 | 252.2 |
| 137 | 141 | 129 | 134 | 135.1 | 270.4 |
| 121 | 143 | 137 | 128 | 131.9 | 263.9 |
| 118 | 124 | 128 | 119 | 122.1 | 244.3 |
| 126 | 132 | 111 | 136 | 125.8 | 251.7 |
| 110 | 134 | 128 | 117 | 121.8 | 243.7 |
| 122 | 120 | 119 | 133 | 123.3 | 246.7 |
| 116 | 134 | 125 | 109 | 120.6 | 241.2 |
| 109 | 137 | 123 | 118 | 121.3 | 242.6 |
| 127 | 138 | 120 | 116 | 124.9 | 249.9 |
| 138 | 110 | 145 | 103 | 122.7 | 245.4 |
| 107 | 115 | 117 | 135 | 118.0 | 236.1 |
| 122 | 129 | 135 | 116 | 125.2 | 250.6 |

| **weight** | **Spores per**  **pot count** | **spores per**  **plant count** | **Spores per plant**  **hemocytometer**  **standardized** |
| --- | --- | --- | --- |
| 68 | 14469.7 | 3617.4 | 36174437.9 |
| 68 | 14102.7 | 3525.6 | 35256977.6 |
| 68 | 13344.2 | 3336.1 | 33360645.4 |
| 68 | 15212.4 | 3803.1 | 38031009.2 |
| 68 | 15079.8 | 3769.9 | 37699596.4 |
| 68 | 15096.5 | 3774.1 | 37741416.4 |
| 68 | 16270.9 | 4067.7 | 40677359.8 |
| 68 | 14633.9 | 3658.4 | 36584821.0 |
| 68 | 14845.9 | 3711.5 | 37114991.6 |
| 68 | 17083.2 | 4270.8 | 42708087.5 |
| 68 | 16908.8 | 4227.2 | 42272014.9 |
| 68 | 16767.9 | 4191.9 | 41919967.8 |
| 68 | 15745.3 | 3936.3 | 39363438.0 |
| 68 | 16134.1 | 4033.5 | 40335244.0 |
| 68 | 16578.5 | 4144.6 | 41446483.0 |
| 68 | 16554.8 | 4138.7 | 41387076.5 |
| 68 | 15417.6 | 3854.4 | 38544093.7 |
| 68 | 16056.3 | 4014.1 | 40140908.9 |
| 68 | 16536.7 | 4134.1 | 41341761.1 |
| 68 | 16005.1 | 4001.2 | 40012829.6 |
| 68 | 16067.4 | 4016.8 | 40168577.2 |
| 68 | 18288.3 | 4572.1 | 45720834.6 |
| 68 | 17299.1 | 4324.8 | 43247975.3 |
| 68 | 15592.5 | 3898.1 | 38981328.7 |
| 68 | 17113.6 | 4278.4 | 42784080.5 |
| 68 | 17155.0 | 4288.7 | 42887592.6 |
| 68 | 17268.0 | 4317.0 | 43170010.9 |
| 68 | 16571.3 | 4142.8 | 41428301.1 |
| 68 | 17094.6 | 4273.6 | 42736556.6 |
| 68 | 16246.4 | 4061.6 | 40616248.5 |
| 68 | 16233.5 | 4058.3 | 40583876.3 |
| 68 | 17139.9 | 4284.9 | 42849785.2 |
| 68 | 16385.4 | 4096.3 | 40963635.9 |
| 68 | 16100.7 | 4025.1 | 40251868.7 |
| 68 | 15363.5 | 3840.8 | 38408990.0 |
| 68 | 17454.4 | 4363.6 | 43636023.5 |
| 68 | 18174.5 | 4543.6 | 45436465.6 |
| 68 | 16693.2 | 4173.2 | 41732962.5 |
| 68 | 17155.6 | 4288.9 | 42889088.4 |
| 68 | 18384.3 | 4596.1 | 45960787.8 |
| 68 | 17949.4 | 4487.3 | 44873702.4 |
| 68 | 16617.1 | 4154.2 | 41542638.2 |
| 68 | 17119.4 | 4279.8 | 42798693.1 |
| 68 | 16577.2 | 4144.3 | 41443101.2 |
| 68 | 16779.3 | 4194.8 | 41948248.9 |
| 68 | 16406.4 | 4101.6 | 41016041.3 |
| 68 | 16501.5 | 4125.3 | 41253657.9 |
| 68 | 16996.8 | 4249.2 | 42492220.6 |
| 68 | 16688.1 | 4172.0 | 41720371.5 |
| 68 | 16057.9 | 4014.4 | 40144793.5 |
| 68 | 17040.2 | 4260.0 | 42600455.4 |
