# Supplementary figures and images for: A clubroot pathogen effector targets cruciferous cysteine proteases to suppress plant immunity
Source: Virulence. 2021 Sep 13;12(1):2327–40. doi: 10.1080/21505594.2021.1968684 (PMC8451464; doi:10.1080/21505594.2021.1968684)

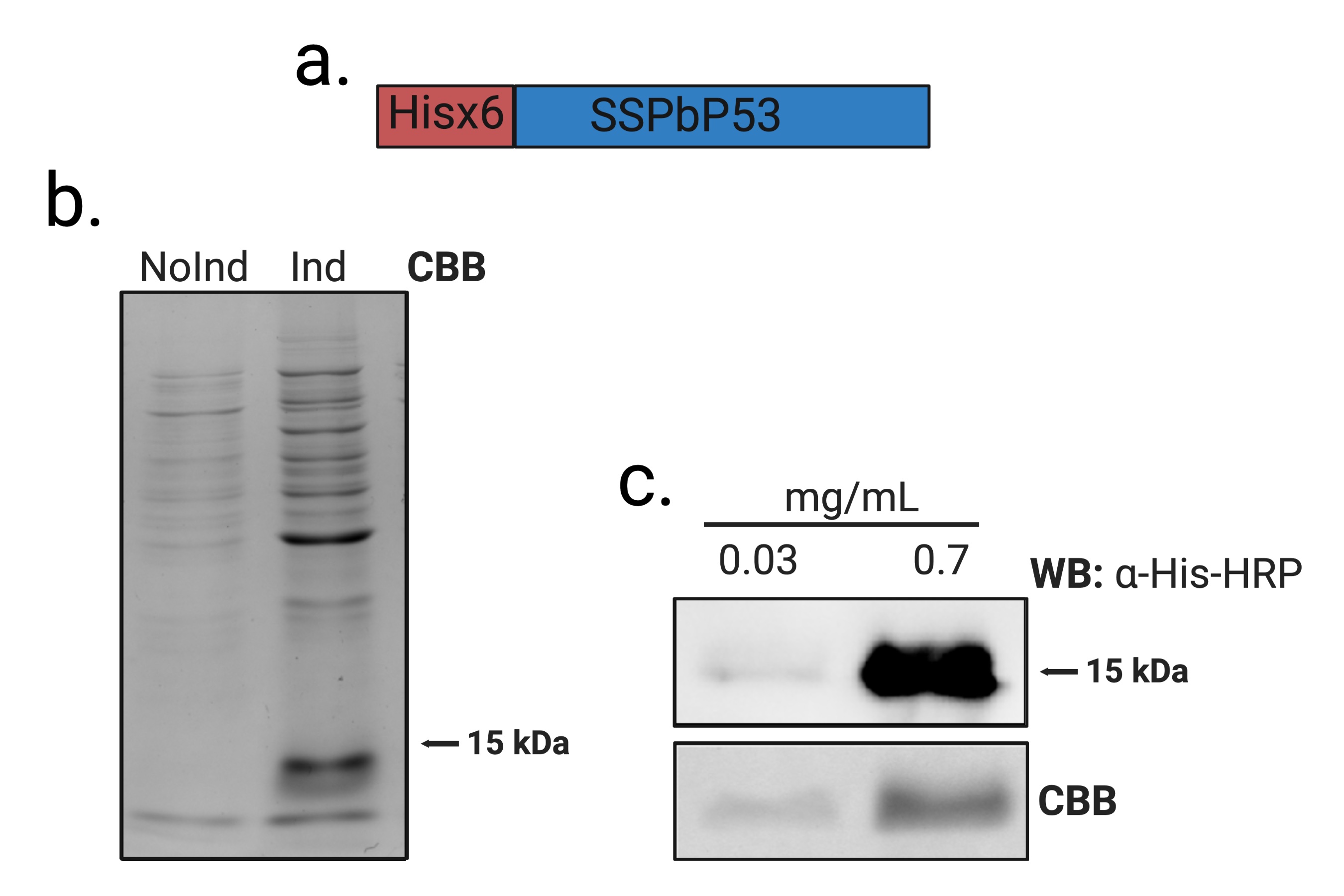

Supplement: Supplemental Material [file KVIR_A_1968684_SM3048.zip › suppll/Fig. S1.jpg]

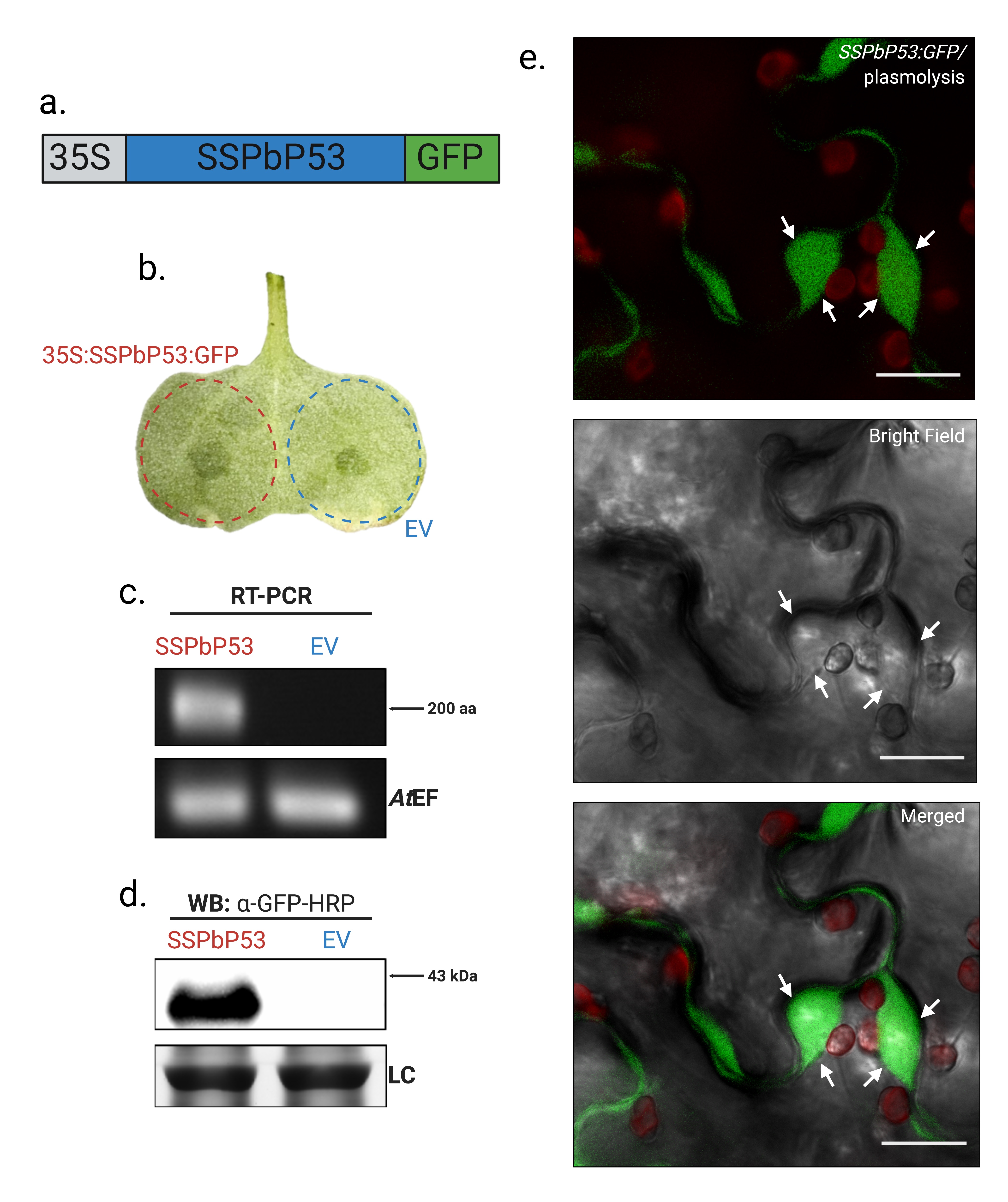

Supplement: Supplemental Material [file KVIR_A_1968684_SM3048.zip › suppll/Fig. S2.jpg]

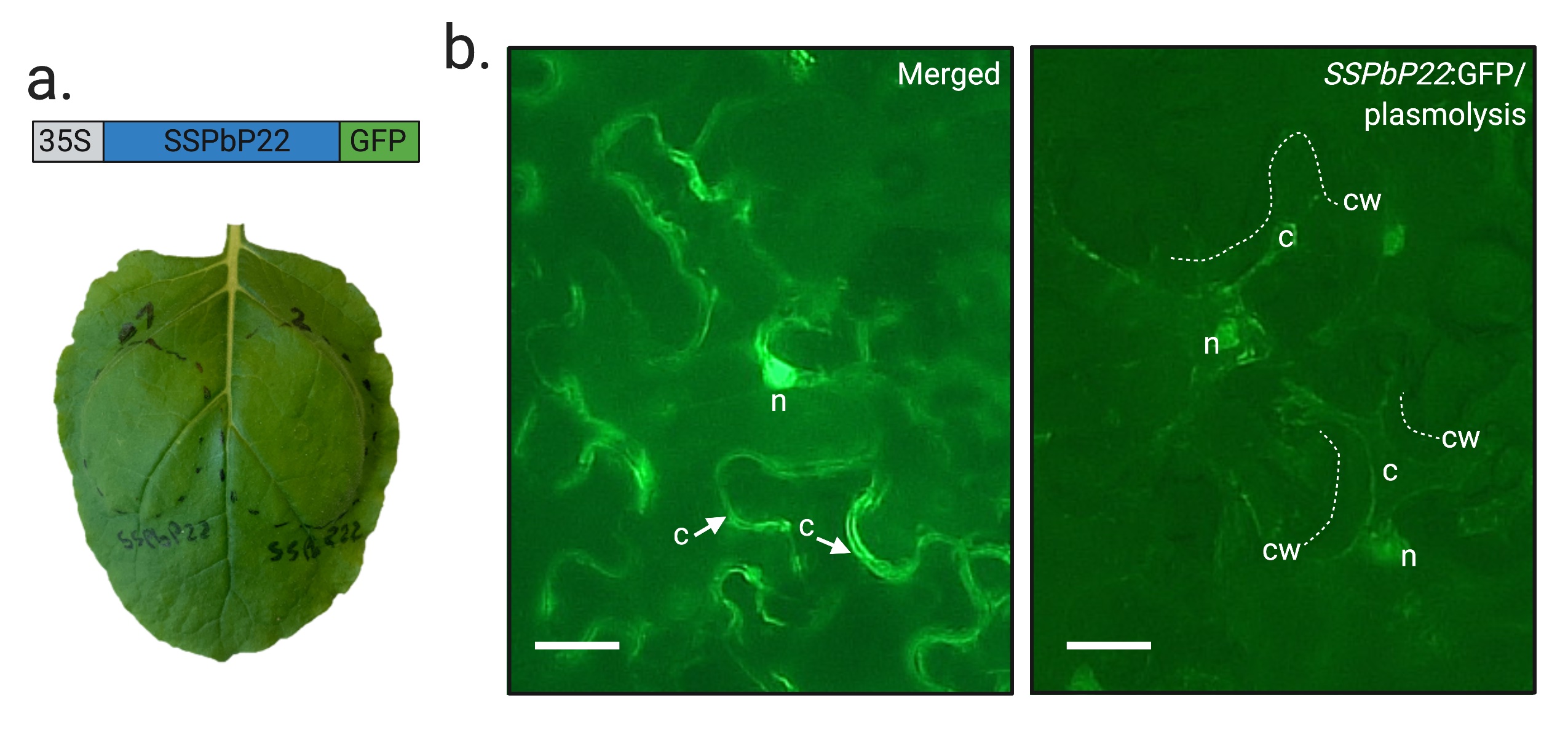

Supplement: Supplemental Material [file KVIR_A_1968684_SM3048.zip › suppll/Fig. S3.jpg]

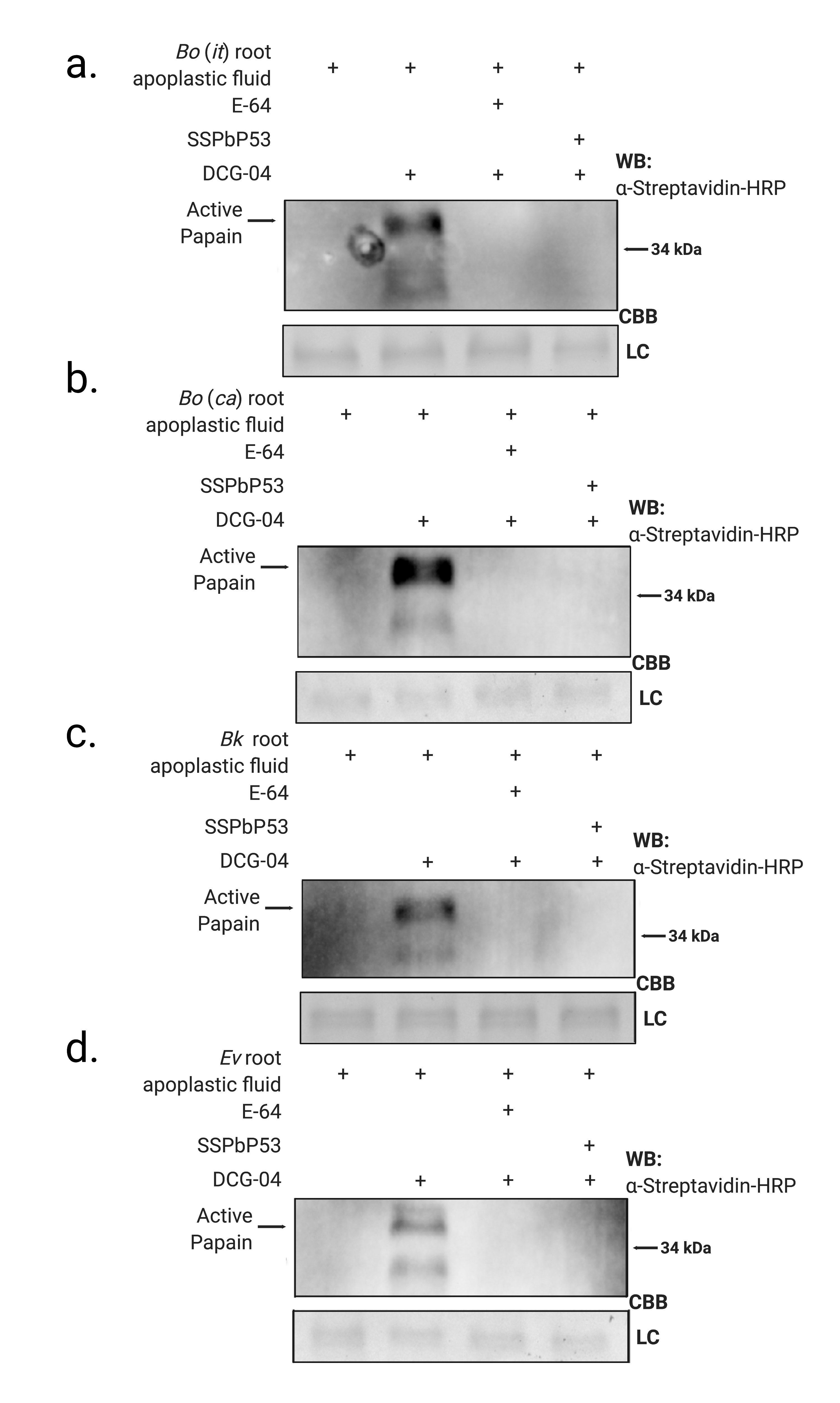

Supplement: Supplemental Material [file KVIR_A_1968684_SM3048.zip › suppll/Fig. S4.jpg]

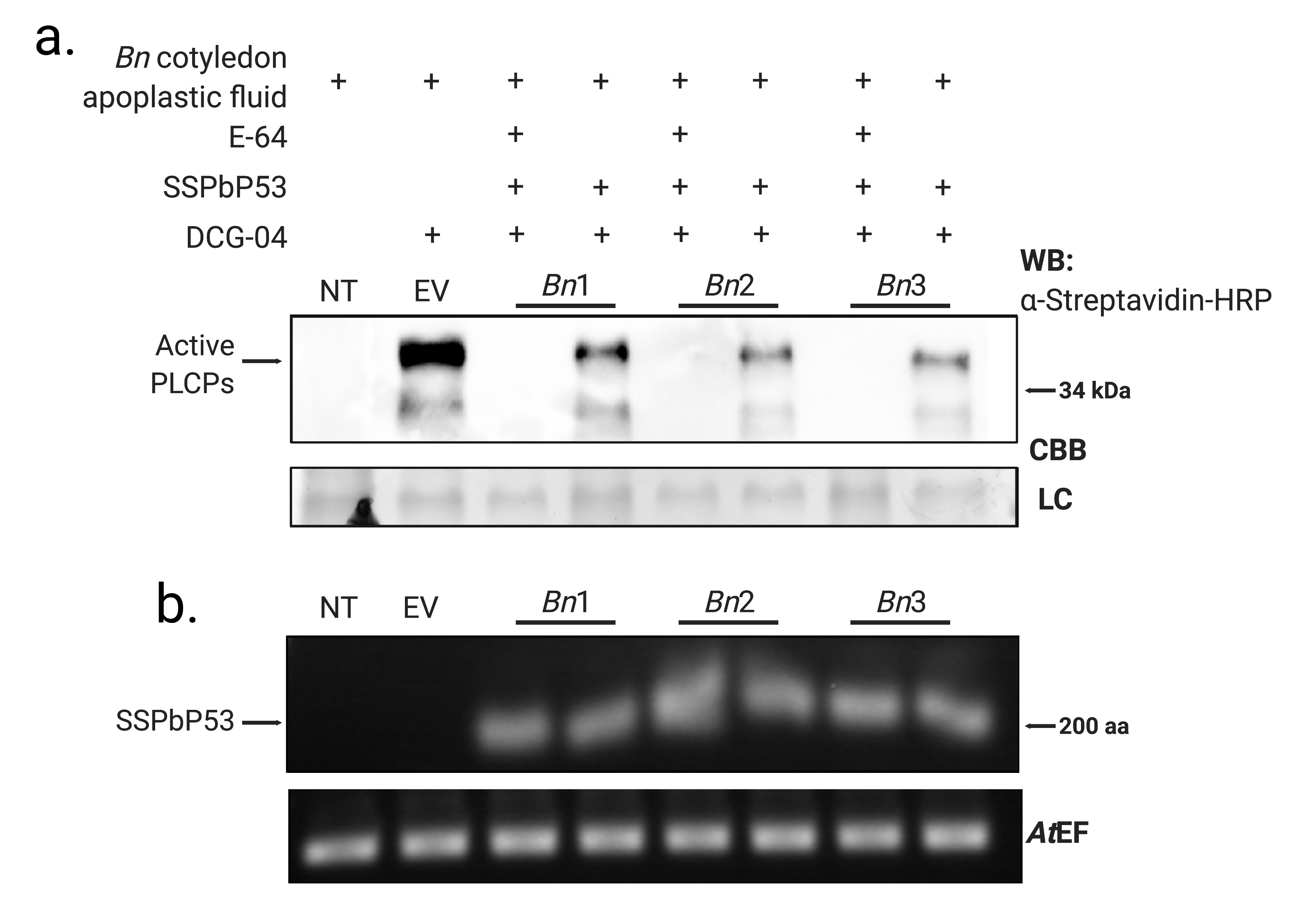

Supplement: Supplemental Material [file KVIR_A_1968684_SM3048.zip › suppll/Fig. S5.jpg]

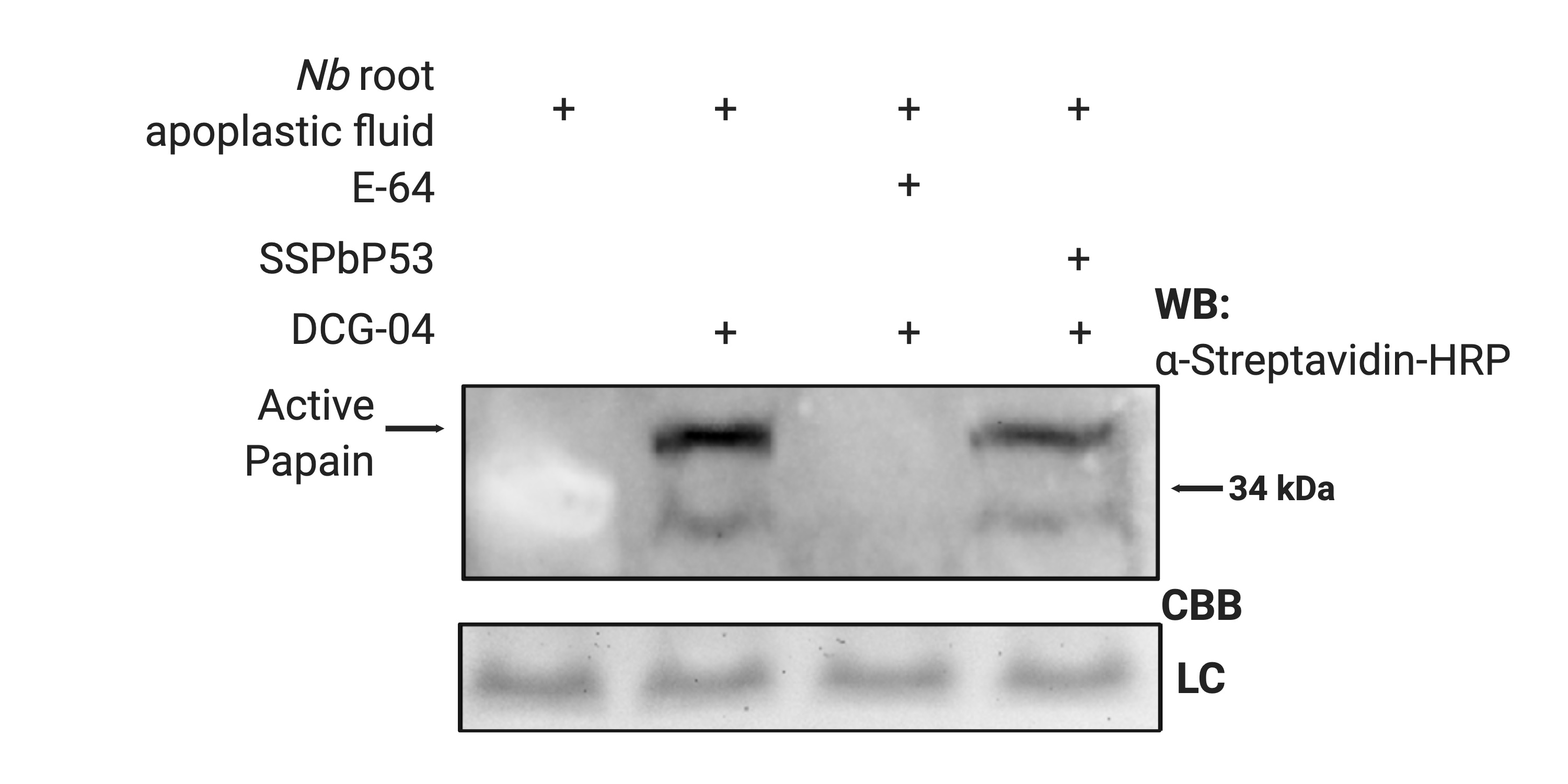

Supplement: Supplemental Material [file KVIR_A_1968684_SM3048.zip › suppll/Fig. S6.jpg]

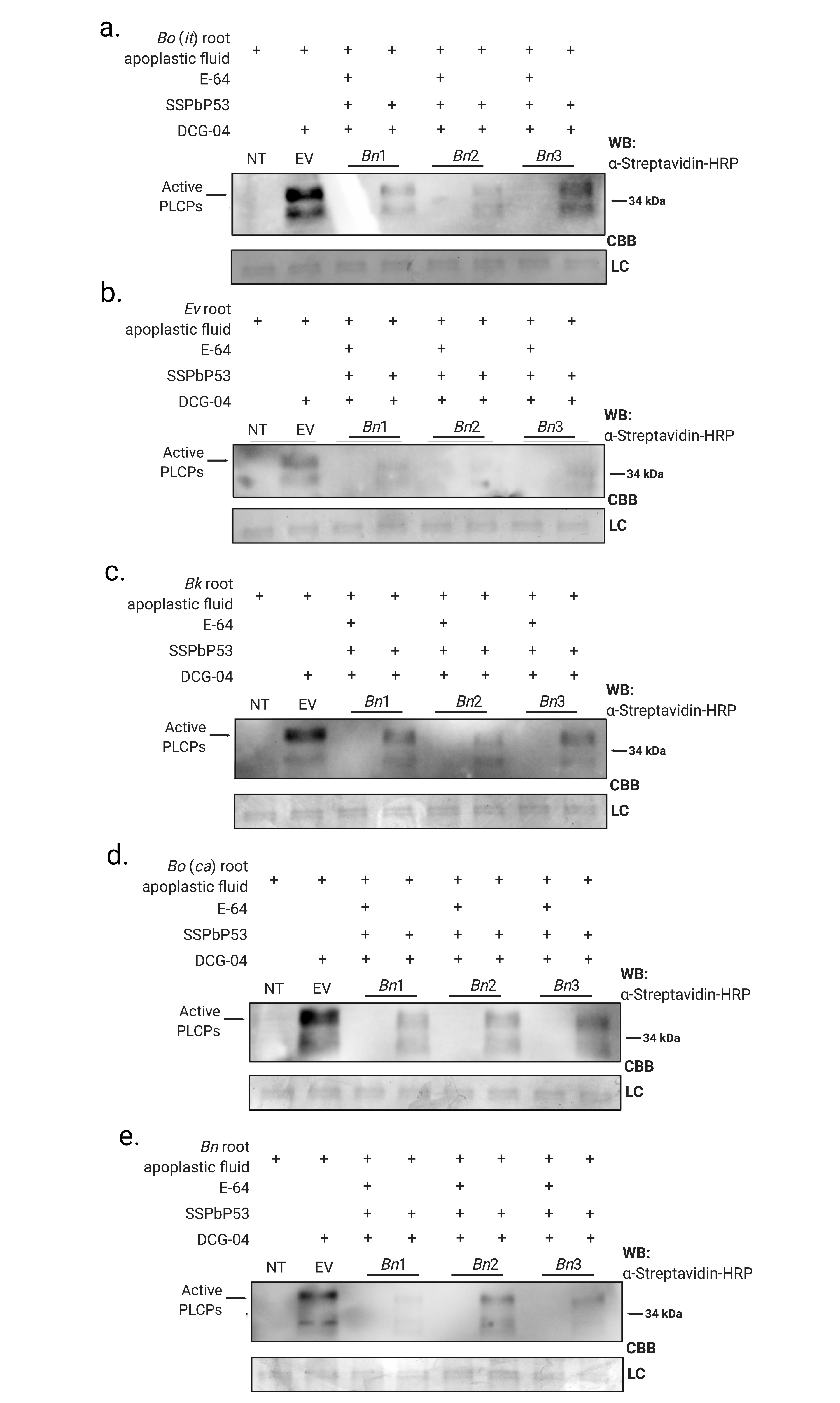

Supplement: Supplemental Material [file KVIR_A_1968684_SM3048.zip › suppll/Fig. S7.jpg]

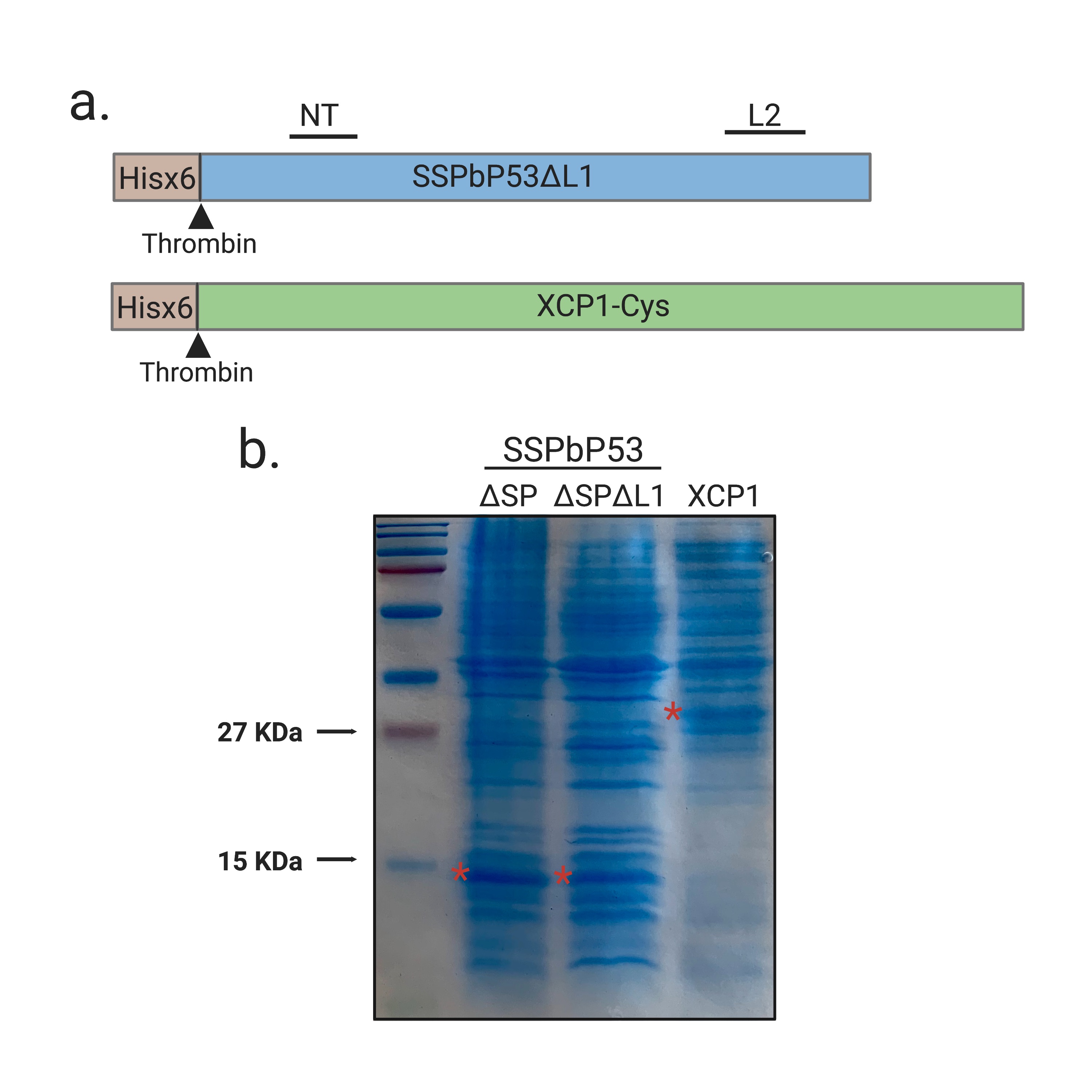

Supplement: Supplemental Material [file KVIR_A_1968684_SM3048.zip › suppll/Fig. S8.jpg]

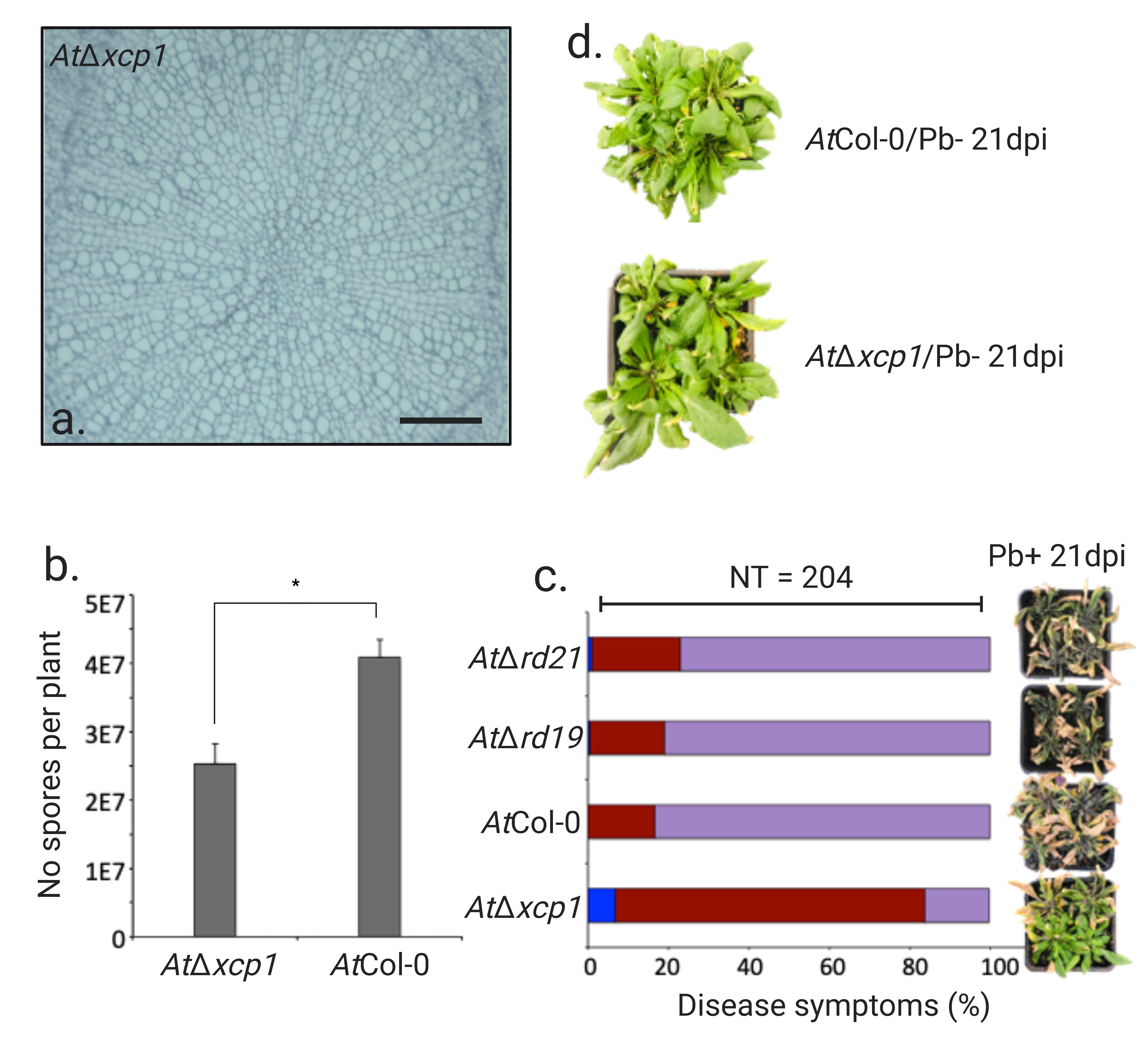

Supplement: Supplemental Material [file KVIR_A_1968684_SM3048.zip › suppll/Fig._S9.jpg]
